# CgII cleaves DNA using a mechanism distinct from other ATP-dependent restriction endonucleases

**DOI:** 10.1093/nar/gkx580

**Published:** 2017-07-07

**Authors:** Paulius Toliusis, Mindaugas Zaremba, Arunas Silanskas, Mark D. Szczelkun, Virginijus Siksnys

**Affiliations:** 1 Department of Protein–DNA Interactions, Institute of Biotechnology, Vilnius University, Sauletekio al. 7, LT-10257, Vilnius, Lithuania; 2 DNA–Protein Interactions Unit, School of Biochemistry, Biomedical Sciences Building, University of Bristol, Bristol, BS8 1TD, UK

## Abstract

The restriction endonuclease CglI from *Corynebacterium glutamicum* recognizes an asymmetric 5′-GCCGC-3′ site and cleaves the DNA 7 and 6/7 nucleotides downstream on the top and bottom DNA strands, respectively, in an NTP-hydrolysis dependent reaction. CglI is composed of two different proteins: an endonuclease (R.CglI) and a DEAD-family helicase-like ATPase (H.CglI). These subunits form a heterotetrameric complex with R_2_H_2_ stoichiometry. However, the R_2_H_2·_CglI complex has only one nuclease active site sufficient to cut one DNA strand suggesting that two complexes are required to introduce a double strand break. Here, we report studies to evaluate the DNA cleavage mechanism of CglI. Using one- and two-site circular DNA substrates we show that CglI does not require two sites on the same DNA for optimal catalytic activity. However, one-site linear DNA is a poor substrate, supporting a mechanism where CglI complexes must communicate along the one-dimensional DNA contour before cleavage is activated. Based on experimental data, we propose that adenosine triphosphate (ATP) hydrolysis by CglI produces translocation on DNA preferentially in a downstream direction from the target, although upstream translocation is also possible. Our results are consistent with a mechanism of CglI action that is distinct from that of other ATP-dependent restriction-modification enzymes.

## INTRODUCTION

**Figure 1.**
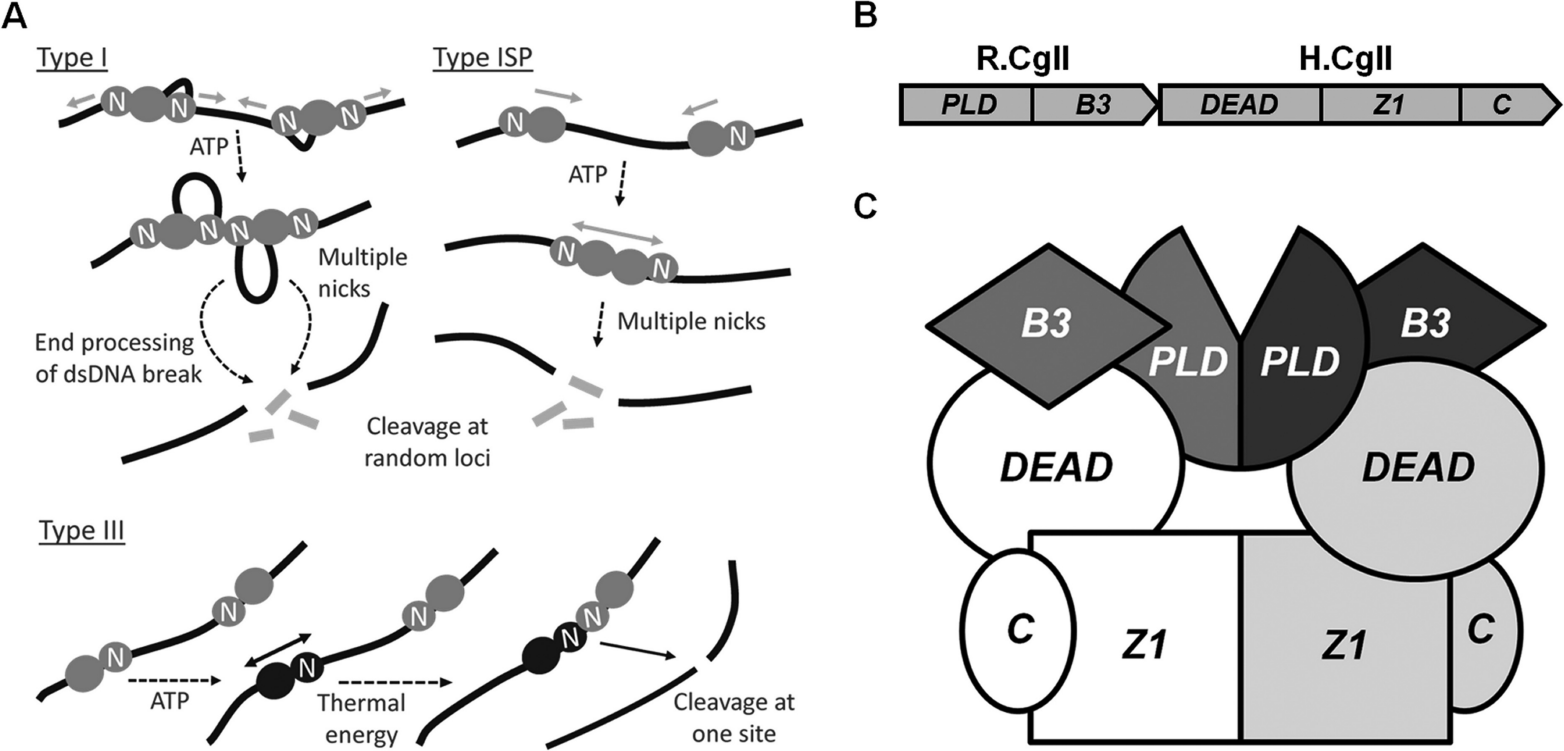
The adenosine triphosphate (ATP)-dependent restriction-modification (RM) enzymes. (**A**) Mechanisms of DNA cleavage by Type I, ISP and III RM enzymes. Nuclease subunit/domains are labeled ‘N’. For Type I enzymes, each complex has two helicase–nuclease subunits which can translocate away each side of the site, drawing in two DNA loops (only one loop is shown per complex for clarity). Upon collision of two converging translocating enzymes at a non-specific site, the nucleases engage and generate a dsDNA break (2). Additional processing leads to DNA shortening and release of small fragments (54,55). For Type ISP enzymes, translocation is unidirectional. Upon collision at a non-specific site, the nuclease domains are held apart. It is proposed that movement of the collision complex results in multiple nicks that generate the dsDNA break (9,40). For the Type III enzymes, ATP hydrolysis leads to a conformation switch into a DNA sliding state. Long-range communication is bidirectional and driven by thermal energy. Upon collision with a second enzyme complex bound to a target site, the nucleases engage and generate a dsDNA break (18). (**B**) Domain organization of the R- and H.CglI proteins. R.CglI (NCgl1704) is a putative REase with the phospholipase D (PLD)-superfamily nucleolytic and B3-like DNA binding domain. H.CglI (NCgl1705) is predicted superfamily 2 (SF 2) helicase/ATPase containing uncharacterized Z1-superfamily and C-terminal domains. (**C**) Proposed model for the R_2_H_2_·CglI complex. R.CglI is composed of the PLD and B3 domains (colored gray and dark gray). H.CglI contains the DEAD, Z1 and C-terminal domains (colored white and light gray). Figure was made according to (28).

Restriction-modification (RM) enzymes are important player in protecting bacterial cells against invasion by foreign DNA (1–3). The Types I, II and III RM enzymes are composed of two main enzyme activities: an endonuclease and a methyltransferase. The methyltransferases methylate DNA targets and protect host genomes from self-cleavage by the cognate endonucleases. Foreign DNA that has unmodified targets will be degraded by the introduction of double strand DNA breaks by the endonuclease. Type II restriction enzymes differ from Type I and III restriction enzymes in several ways. A principal difference is that they do not require adenosine triphosphate (ATP) for DNA cleavage (4). In contrast, all Type I, Type for single polypeptide (ISP) and Type III RM enzymes studied to-date use ATP hydrolysis for a long-range communication between enzymes along the one-dimensional (1D) contour of the DNA (5–9). The protein–protein interaction between communicating subunits activates DNA cleavage. In some instances the relative orientation of the asymmetric targets is also important (called ‘site orientation preference’). Examination of the communication mechanism and of the effect of different arrangements of targets on DNA cleavage activity can distinguish between the different mechanisms:

- For the classical Type I RM enzymes, there are two independent motors subunits (HsdR) that catalyze stepwise translocation along the DNA up- and downstream from the site; i.e. translocation is simultaneously bidirectional (Figure 1A) (10–12). Each motor consumes at least one ATP to move 1 bp and translocation can be measured using the triplex displacement assay (10,13). DNA cleavage occurs at random non-specific sites when two motors collide along the 1D DNA contour (forming a ‘collision complex’). On a plasmid this can be achieved by two motors from the same complex and so a single site is sufficient to produce cleavage. However, on linear substrate at least two DNA sites are needed; with a single site each motors run to the DNA ends and cannot collide. The Type I target sites are asymmetric but since translocation is bidirectional, there is absolutely no effect of the relative orientation of pairs of sites on the efficiency or rate of DNA cleavage (i.e. there is no site orientation preference) (10).
- For the single polypeptide Type ISP RM enzymes, there is only a single motor that moves the protein downstream from the asymmetric target site; i.e. translocation is strictly unidirectional (Figure 1A) (14). The motor consumes at least one ATP to move 1 bp and translocation can be measured using the triplex displacement assay (14–16). DNA cleavage by the translocating motor is activated by collision with another Type ISP enzyme. On one site plasmid or linear DNA with sites in direct repeat, the collision is described as a ‘rear-end’ collision and only inefficient cleavage of one strand (nicking) results. The nicking is located at a target site where the collision occurred. On linear DNA with sites in inverted repeat, efficient dsDNA cleavage is activated when the enzymes can collide ‘head-on’. This can only occur where target sites are in head-to-head orientation. Linear DNA with targets in tail-to-tail orientation never produces collision events as the enzymes translocate away from one another (14–17).
- For the Type III RM enzymes, there is a single ATPase subunit (Res) that undergoes a small burst of ATP hydrolysis (10–30 ATPs) while the enzyme is at the site (Figure 1A). This produces a conformation switch into a ‘DNA sliding’ conformation (18). When the enzyme subsequently dissociates from the site, it can move both up- and downstream driven purely by thermal motion (19). This motion is independent of ATP hydrolysis and does not produce triplex displacement (in fact the triplex can be by-passed) (20). The original binding orientation of the enzyme is maintained during the sliding and thus cleavage shows site orientation preference (5,8,21). On plasmid or linear DNA with sites in inverted repeat (either head-to-head or tail-to-tail), dsDNA cleavage can be activated by head-on collision between a sliding enzyme and an enzyme bound to a site (5,20,22). The efficiency of cleavage of linear DNA relative to circular DNA is poor as the sliding enzymes can dissociate via the DNA ends of the former (5). Additionally, the relative cleavage efficiency of tail-to-tail sites on linear DNA can be lower if the enzyme binds tightly to its site (22). For the single site DNA or two site head-to-tail (HtT) DNA, in either linear or circular form, DNA cleavage is not observed as all collisions are in the incorrect ‘rear-end’ orientation and the nuclease domains cannot engage (5,20).

In addition to their important biological roles as innate immune systems against DNA invasion (23), RM enzymes are model nucleoprotein machines and study of their mechanisms have added significantly to fundamental knowledge about DNA binding, DNA methylation and DNA cleavage (e.g. (24–26). For the ATP-dependent RM enzymes, there have been the additional opportunities to study how helicase-like motors utilize ATP hydrolysis for non-classical DNA translocation and switching roles (as noted above). These mechanisms have parallels to ATP-dependent motors in chromatin remodeling (27) and in mismatch repair (6). Previously, it was shown that the CglI enzyme has properties that differ from all Types of RM systems and cannot be readily assigned to any particular type (28). Examining these properties in more detail will add to our understanding of the diversity of roles for helicase-like motors.

CglI requires hydrolysis of ATP (or other nucleotide triphosphates) to produce cleavage of linear bacteriophage DNA with multiple sites in different relative orientations (28). The level of ATP hydrolysis (∼170 ATP/s/monomer) is consistent with a stepwise translocation mechanism rather than a Type III-like conformational switch/sliding mechanism (28). The stoichiometry of the CglI enzyme is R_2_H_2_ and each complex has one complete nuclease active site sufficient to cut one DNA strand, and two helicase-like motor domains and DNA binding domains (Figure 1B and C) (28). Therefore both uni- and bidirectional communication are possible. It is also likely that two R_2_H_2_ complexes will be required to produce a single dsDNA break since dimerization of the R-subunits will produce only a single active site, akin to BfiI (29). This would also predict a requirement for inter-target communication. Each R_2_H_2_ complex also contains two B3-like DNA binding domains, which also offers the possibility of communication by looping between pairs of targets.

In this paper, we report the investigation of the CglI catalytic mechanism of DNA cleavage. We demonstrate that CglI efficiently hydrolyzes one-site circular DNA and does not strictly require the presence of a second DNA target *in cis* on the same DNA. However, two-site linear DNA substrates can be cleaved more efficiently than one-site linear DNA substrates. Moreover, on HtT linear DNA, cleavage predominantly occurs at one of the two targets. These cleavage data are consistent with a communication scheme that follows the 1D DNA contour with some directional bias. Using a triplex displacement assay, we confirm that ATP-dependent translocation from a target has partial directionality, with a preference for communication downstream from the asymmetric recognition sequence (5′-GCCGC-3′). We also demonstrate that CglI has the unexpected ability to displace a triplex *in trans*, in a reaction that does not require entry/exit from DNA ends. We demonstrate that *in trans* triplex displacement occurs via simultaneous binding of both DNA molecules (specific and non-specific). On the basis of the DNA cleavage and triplex displacement experiments, we propose that CglI translocates bidirectionally along the 1D DNA contour from its target site and cleaves dsDNA when two R_2_H_2_·CglI complexes collide with each other (one translocating and one bound to a target site). In some cases the translocating species can be delivered *in trans* having initiated on a separate DNA molecule. This reaction scheme is quite distinct to that observed for Type I, ISP or III RM enzymes, and adds further to our understanding of the remarkable diversity of mechanisms that have evolved to combat bacteriophage infection.

## MATERIALS AND METHODS

### Expression and purification of CglI proteins

The wt CglI and its active site mutants were expressed and purified as described earlier (28).

### Oligonucleotides and DNA

Named oligonucleotide sequences are given in Supplementary Table S1. Plasmid substrates and templates are summarized in Supplementary Table S2.

#### Substrates for DNA cleavage experiments

One-site open-circle (OC) and one-site full-length linear (FLL) substrates were obtained from one-site supercoiled circular (SC) p1 plasmid DNA by cleavage of a single DNA strand with a Cas9(H840A)–crRNA complex and linearization with restriction endonuclease NdeI, respectively. The HtN fragment used in DNA cleavage stimulation experiments was obtained from one-site SC p1 DNA by linearization with restriction endonuclease XhoI and labeling of 5′ overhangs with [γ^33^P]ATP (Perkin Elmer) and T4 polynucleotide kinase (Thermo Fisher Scientific, Vilnius, Lithuania). The HtT1 and HtT2 fragments were obtained from two-site SC p2_HtT DNA by linearization with restriction endonucleases XhoI and NdeI, respectively. HtH and HtT fragments were obtained from two-site SC p2_TtT DNA by linearization with restriction endonucleases XhoI and NdeI, respectively. A total of 85-bp fragment S85 containing a single target of CglI was synthesized by polymerase chain reaction (PCR) using MZ-318 and AS-41 oligonucleotides as primers and pECFP-ICAD-S(NLS) plasmid as a template (Supplementary Tables S1 and 2). All linear and OC fragments were purified using the GeneJET™ PCR Purification Kit (Thermo Fisher Scientific, Vilnius, Lithuania).

#### Substrates for triplex displacement assay

Three different 278 bp substrates N, H and T were synthesized by PCR using MZ-503 and MZ-504 oligonucleotides as primers and pRMA03_0, pRMA03_1 and pRMA03_1_IN plasmids as templates (14), respectively (Supplementary Tables S1 and 2). 5′-end biotinylated 278 bp substrate N was obtained from the previously synthesized substrate N by PCR using MZ-948 and MZ-504 oligonucleotides (Supplementary Table S1). A total of 3383 bp CN plasmid substrate was constructed by ligation of the 278 bp N fragment into p0s vector pre-digested with restriction endonuclease EheI. A total of 3383 bp LN substrate was generated from CN plasmid by linearization with restriction endonuclease CaiI. L0 and L1 were obtained from p0s and p1s plasmid linearization with restriction endonuclease NdeI, respectively. All PCR generated fragments were purified using the GeneJET^™^ PCR Purification Kit (Thermo Fisher Scientific, Vilnius, Lithuania). The 5′-end of triplex forming oligonucleotide (TFO) was labeled with [γ^33^P]ATP (Perkin Elmer) and T4 polynucleotide kinase (Thermo Fisher Scientific, Vilnius, Lithuania).

### DNA cleavage assay

DNA (10 nM) cleavage reactions were performed at 37°C in Reaction Buffer 1 (33 mM Tris-acetate (pH 7.0 at 25°C); 20 mM Mg-acetate; 0.1 mg/ml bovine serum albumin (BSA); 1 mM dithiothreitol (DTT); 4 mM ATP; 20 mM phosphocreatine and 5 U/ml creatine kinase). Reactions were initiated by addition of 1000 nM H.CglI and 1000 nM R.CglI (in terms of monomer) and stopped by addition of 2× Loading solution (75 mM ethylenediaminetetraacetic acid (EDTA), 0.6% (w/v) orangeG, 50% (v/v) glycerol, 0.5% (w/v) sodium dodecyl sulfate (SDS), pH 8.0 at 25°C) and incubation at 70°C for 15 min. The reaction products were separated by electrophoresis through 0.8 or 1.0% (w/v) agarose gels with 0.5 μg/ml ethidium bromide and the relative amount of dsDNA cleavage quantified by gel densitometry using BioDocAnalyze image analyzer (Biometra, Göttingen, Germany) and the images analyzed with OptiQuant software (Packard Instrument). To determine whether the ring size of one-site circular DNA has an effect on cutting efficiency of CglI one-site plasmid DNAs with various ring sizes (p1_2064, p1_3105, p1_4320, p1_4498, p1_5722, Supplementary Table S2) were incubated with 1000 nM H.CglI and 1000 nM R.CglI (in terms of monomer) at 25°C in Reaction Buffer 1 (indicated above). Reactions were stopped and products analyzed as described above.

To determine the DNA cleavage position of CglI pHtT or pTtT, plasmid DNA (10 nM) was incubated with 1000 nM H.CglI and 1000 nM R.CglI (in terms of monomer) for 1 h at 25°C in Reaction Buffer 1 (indicated above). Reactions were stopped as mentioned above. Linearized plasmids were purified following agarose gel electrophoresis using GeneJET Gel Extraction Kit (Thermo Fisher Scientific, Vilnius, Lithuania). The linear purified DNAs were sequenced using MZ-438 and MZ-437 oligonucleotides as primers (Supplementary Table S1).

DNA cleavage stimulation reactions contained 1 nM of the HtN fragment and were performed at 20°C in Reaction Buffer 2 (33 mM Tris-acetate (pH 7.0 at 25°C); 10 mM Mg-acetate; 0.1 mg/ml BSA; 1 mM DTT; 4 mM ATP; 150 mM KAc; 20 mM phosphocreatine and 5 U/ml creatine kinase) and in the presence of 10, 50, 100 or 250 nM of the S85 fragment. Reactions were initiated by addition of 1000 nM H.CglI and 1000 nM R.CglI (in terms of monomer) and stopped as mentioned above. The products were separated by electrophoresis through 1.0% (w/v) agarose gels in 40 mM Tris, 20 mM acetic acid and 1 mM EDTA (pH 8.3 at 25°C). Gels were air-dried and scanned in Fujifilm FLA-5100 fluorescent image analyzer (Fujifilm, Tokyo, Japan) and the image analyzed with OptiQuant software (Packard Instrument) to determine the volume of each band, taking into account background readings. The fraction of DNA substrate left in each sample was calculated as volume_(DNA substrate left)_/(volume_(DNA substrate left)_ + volume_(DNA product)_).

### Triplex formation and analysis

A total of 50 nM linear DNA and 25 nM 5′-end ^33^P-labeled TFO (for *in trans*: 10 nM linear or plasmid DNA and 5 nM ^33^P-labeled TFO) were mixed in buffer MM (10 mM 2-(N-morpholino)ethanesulfonic acid (MES) (pH 5.5 at 25°C), 12.5 mM MgCl_2_) and incubated at 20°C overnight. The triplexes were stored on ice and then diluted 1/10 into Reaction Buffer 1 before use. To analyze the proportion of bound and free TFO, reactions were quenched using 1:1 GSMB buffer (15% (w/v) glucose, 3% (w/v) SDS, 250 mM 3-(N-morpholino)propanesulfonic acid (MOPS) (pH 5.5 at 25°C), 0.4 mg/ml bromophenol blue) and analyzed in 6 or 8% (w/v) polyacrylamide gels (29:1 acrylamide/bisacrylamide in 40 mM Tris-acetate (pH 5.5 at 25°C), 5 mM Na-acetate, 1 mM MgCl_2_) at ∼10 V/cm for 2 h at 4°C or 1% (w/v) agarose gels (40 mM Tris-acetate (pH 5.5 at 25°C), 5 mM Na-acetate, 1 mM MgCl_2_) at ∼8 V/cm for 1 h at 4°C. Gels were air-dried and scanned in Fujifilm FLA-5100 fluorescent image analyzer (Fujifilm, Tokyo, Japan) and the image analyzed with OptiQuant software (Packard Instrument) to determine the volume of each band, taking into account background readings. The fraction of triplex in each sample was calculated as volume_triplex_/(volume_triplex_ + volume_free TFO_) (10).

### CglI translocation reactions

Triplex displacement reactions were performed at 20°C mixing preformed triplex (see above) in 33 mM Tris-acetate (pH 7.0 at 25°C), 0.1 mg/ml BSA (or 1 mg/ml BSA for reactions with magnetic beads), 1 mM DTT, 10 mM Mg-acetate, 150 mM K-acetate, 4 mM ATP or adenylyl-imidodiphosphate (AMP-PNP), 25 mM phosphocreatine and 5 U/ml creatine kinase. Triplex displacement *in trans* reactions were additionally supplemented with 4, 10 or 50 nM of various dsDNAs. Reactions were initiated by addition of 200 nM H.CglI and 200 nM R.CglI to the reaction mixture. Aliquots were removed at fixed time intervals and the reactions stopped by adding GSMB buffer. Resulting samples were analyzed as above. TFO displacement rates are quoted as the mean of three independent experiments.

### Kinetic data analysis

Quantitative approximation of the rates of TFO displacement and DNA cleavage estimated by fitting the data to first or second degree exponential equations using KyPlot version 2.0 (30). Rate constants (*k*_1_ and *k*_2_) for triplex displacement were calculated using following equations:

(1) \begin{equation*} {\rm{S}} = 100\cdot\exp ( - {k_1}\cdot{\rm {}}t) \end{equation*}

(2) \begin{equation*}{\rm{S}} = [{{\rm{A}}_1}] + [100 - {{\rm{A}}_1}]\cdot\exp ( - {k_1}\cdot{\rm{ }}t)\end{equation*}

(3) \begin{equation*}{\rm{S}} = [{{\rm{A}}_1}]\cdot\exp ( - {k_1}\cdot{\rm {}}t) + [100 - {{\rm{A}}_1}]\cdot\exp ( - {k_2}\cdot{\rm {}}t)\end{equation*}

Where S is the percentage of substrate left, A_1_ and 100−A_1_ are the amplitudes of the first and second phases, respectively, *k*_1_ and *k*_2_ are the rates of the first and second phases, respectively, *t* is the reaction time in seconds.

Rate constants (*k*_1_) for DNA cleavage were calculated using following equations:

(4) \begin{equation*}{\rm{S}} = [{{\rm{A}}_1}]\cdot\exp ( - {k_1}\cdot{\rm {}}t)\end{equation*}

(5) \begin{equation*}{\rm{S}} = [{{\rm{A}}_1}]\cdot\exp ( - {k_1}\cdot{\rm{ }}t) + [{{\rm{A}}_2}]\end{equation*}

(6) \begin{equation*}{\rm{P}} = [{{\rm{A}}_1}]\cdot(1 - \exp ( - {k_1}\cdot{\rm{ }}t))\end{equation*}

Where S is the amount of substrate left (nM), P is the amount of product created (nM), A_1_ is the amplitude of the first phase, A_2_ is the free amplitude, *k*_1_ is the rate of the first phase, *t* is the reaction time in seconds. In all cases the kinetic profiles are actually more complex, with cleavage being preceded by multiple steps. Therefore the exponential fits are to be taken as estimates of the relative rates for comparison purposes, rather than measurements of rate constants that correspond to particular mechanistic steps.

## RESULTS AND DISCUSSION

The stress-sensitive RM system CglI (from Corynebacterium glutamicum) was originally assigned as a Type II RM system but has functional and structural similarities to both Type I and III RM systems (23). Here we investigated the mechanisms of DNA hydrolysis and long-range communication of the CglI restriction enzyme. Throughout this work we utilized reaction conditions where the concentration of CglI is significantly in excess of DNA since these are the condition necessary to observe DNA cleavage (28). Super-saturating protein concentration may be required due to a low specific activity but can also be due to the mechanism of the cleavage pathway; for example both Type I and III RM enzymes have been shown to only work at or above ‘single turnover’ conditions as a consequence of long-range communication mechanisms (31–33). Therefore understanding the cleavage mechanism can in part help explain the necessity for certain reaction conditions; the protein–DNA stoichiometry of CglI will be investigated in detail in a further study.

### CglI uses 1D communication to cut DNA

A basic test of the requirement for single or multiple sites for DNA cleavage that has been widely applied to Type II RM enzymes as well as Type I and III enzymes is to follow endonuclease activity on one- and two-site plasmids, and on DNA catenanes (34–36). Simple RM enzymes which only bind a single target site will cleave a two-site plasmid or catenane only 2-fold faster than one-site plasmid, as the increase in site number does not affect the ability to cleave but merely the chance of cleavage occurring at a site at any given point in time (e.g. (37)). In contrast, enzymes that require simultaneous binding of two targets for an efficient DNA hydrolysis will cleave two-site substrates up to 100-fold faster than one-site substrates (e.g. (37)). The two-site catenanes allow two interacting DNA sites to be on separate rings that are topologically-linked, thus holding the sites in proximity to each other. Long-range communication between the pair of sites on the catenane substrate can only occur by three-dimensional (3D) DNA looping rather than via the 1D DNA contour (e.g. by translocation or DNA sliding). If the reaction occurs by a 3D process, such as DNA looping, the reactions with catenane (both sites are held *in trans*) and two site plasmid (both sites are held *in cis*) will occur with similar efficiency (e.g. (38)). Alternatively, if the communication occurs only by 1D process, the reaction of the catenane will occur with the same efficiency as on one site plasmid (e.g. (36)).

**Figure 2.**
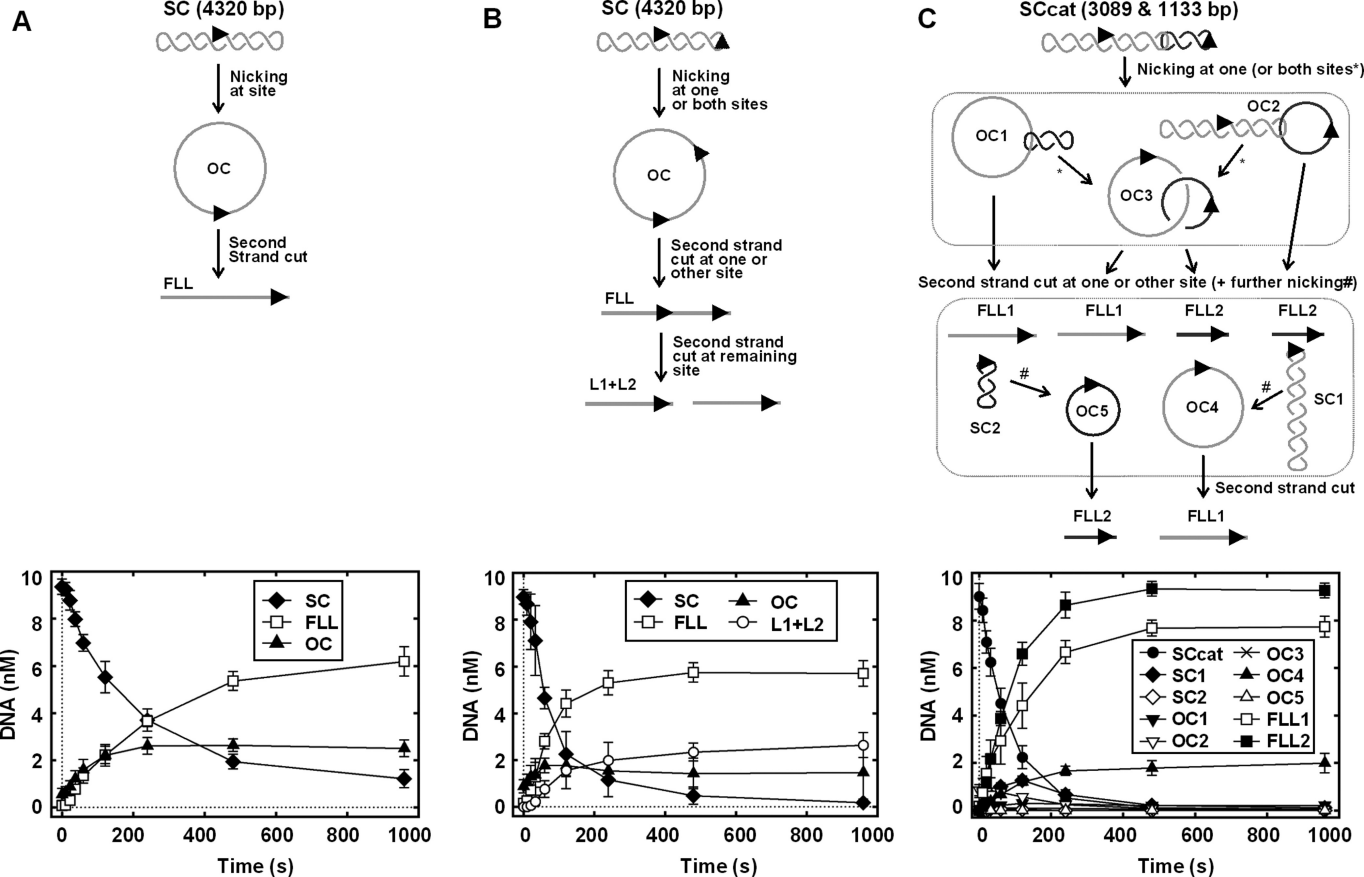
One-site plasmid (**A**), two-site plasmid (**B**) and two-site catenane (**C**) cleavage by CglI. The two-site plasmid substrate was used to form catenane in the presence of Tn21 resolvase (7). The CglI recognition sequence (5′-GCCGC-3′) is shown as an arrowhead (▸). Possible DNA species formed when CglI is incubated with the plasmid or catenane substrates are presented above the graphs: SC—supercoiled circular DNA, OC—open circle DNA (‘nicked’), FLL—full-length linear DNA, L1+L2—linear DNA cut at both CglI sites, SCcat—SC catenane DNA, OC1—catenane nicked in the large ring only, OC2—catenane nicked in the small ring only, OC3—catenane nicked in both rings, OC4—nicked large plasmid, OC5—nicked small ring, FLL1—full-length linear DNA of the large ring, FLL2—full-length linear DNA of the small ring. Note that final FLL products from SCcat can be generated by different routes, requiring either 2, 3 or 4 consecutive independent cleavage events on a single DNA substrate. Reactions contained 10 nM DNA, 4 mM ATP, 500 nM R_2_H_2_·CglI and were conducted as described in ‘Materials and Methods’ section. The rate constant values (5.3 ± 0.3 × 10^−3^ s^−1^ for (A), 1.1 ± 0.1 × 10^−2^ s^−1^ for (B), 1.2 ± 0.1 × 10^−2^ s^−1^ for (C)) were obtained by fitting a single exponential to the time courses of supercoiled form depletion. Points are averages with error bars as standard deviation for at least three repeat reactions.

**Figure 3.**
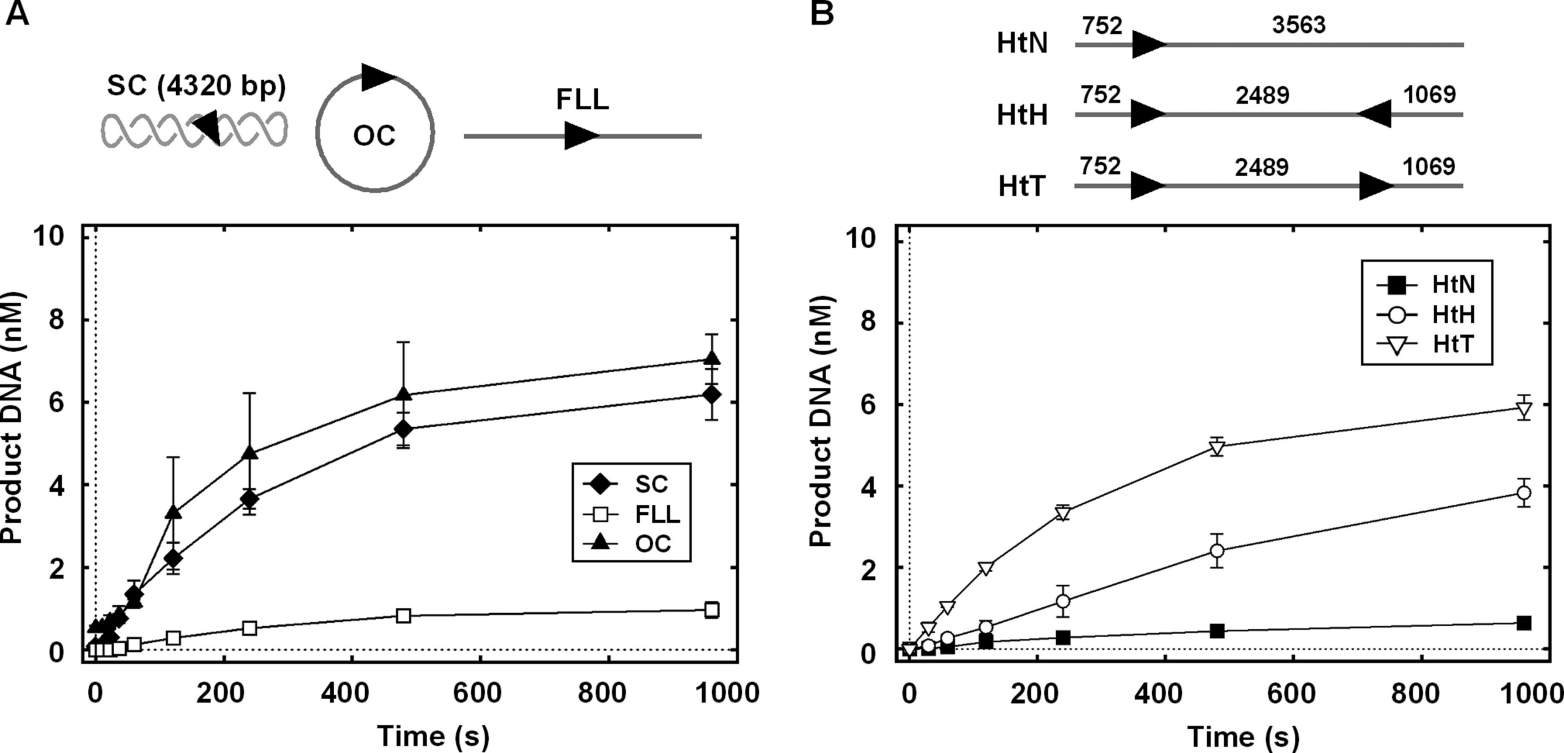
(**A**) Cleavage of the SC, OC and linear one-site plasmid DNA by CglI. Accumulation of the final DNA cleavage products with both DNA strands cleaved at the CglI recognition sequence is shown. The one-site SC DNA was used to form the OC DNA (by cleavage of a single DNA strand with the Cas9–crRNA complex) or the full-length linear DNA (FLL) (both DNA strands were cleaved with the restriction endonuclease NdeI). The CglI recognition sequence (5′-GCCGC-3′) is shown as an arrowhead (▸). The rate constant values (3.6 ± 0.2 × 10^−3^ s^−1^ for SC, 3.0 ± 0.3 × 10^−3^ s^−1^ for FLL and 4.2 ± 0.7 × 10^−3^ s^−1^ for OC) were obtained by fitting a single exponential to the time courses of substrates. (**B**) Cleavage of the linear one- and two-site DNA by CglI. Accumulation of the final DNA cleavage products with both DNA strands cleaved at one CglI recognition sequence is shown. Numbers indicate distances in bp between the CglI targets and DNA ends. The rate constant values (2.0 ± 0.3 × 10^−3^ s^−1^ for HtN, 8.9 ± 1.9 × 10^−4^ s^−1^ for HtH and 3.3 ± 0.1 × 10^−3^ s^−1^ for HtT) were obtained by fitting a single exponential to the time courses of substrates. All reactions contained 10 nM DNA, 4 mM ATP, 500 nM R_2_H_2_·CglI and were conducted as described in ‘Materials and Methods’ section. Points are averages with error bars as standard deviation for at least three repeat reactions.

First, control DNA cleavage experiments were performed using DNA without a target site (zero-site plasmid) (Supplementary Figure S1); only background levels of DNA cleavage were observed, demonstrating the requirement for a specific CglI target site. Next, one-site plasmid DNA, a two-site HtT (HtT orientation of the asymmetric recognition sequence 5′-GCCGC-3′) plasmid DNA and a catenane (generated from the HtT plasmid DNA using the Tn21 resolvase), were used as substrates (Figure 2) (7). The results show that one-site circular DNA is cut only 2-fold slower than the two-site circular DNA, indicating that only one DNA site is necessary for efficient DNA cleavage: the rate of disappearance of the one-site substrate, which represents the first strand break, was only 2-fold slower than the equivalent rate on either of the two-site substrates (Figure 3 and Supplementary Table S3). Note that in all cases the kinetic profiles are actually complex, with cleavage being preceded by multiple steps. Therefore the exponential fits are to be taken as estimates of the relative rates for comparison purposes, rather than measurements of rate constants that correspond to particular mechanistic steps. Additionally we note that the activity of CglI reduces with incubation at 37°C, so rates may slow with time and reactions may not always go to completion.

A simple explanation of the 2-fold difference in the rates (Figure 3 and Supplementary Table S3) is that the initial strand break is twice as likely in the presence of two recognition sites. Disappearance of the two-site catenane substrate band on the gel can be due to cleavage of either the large or small ring. Similarly, the two-site plasmid substrate band can disappear due to cleavage at one or other of the sites. In comparison, disappearance of the one-site plasmid substrate can only occur due to cleavage at one site. Therefore a non-communicating system might show a 2-fold difference between one-site and substrates, regardless of topology. This does not rule out long-range communication, but for CglI a second site *in cis* (plasmid) or *in trans* (catenane) does not necessarily have a large activating effect on DNA cleavage of the circular DNA substrates, where one site can communicate through 1D space with another molecule bound at the same site.

We note that cleavage of the two rings of the two-site catenane to FLL products is more efficient than the two-site plasmid (Figure 2B and C). While the efficiency appears to scale with size of the DNA rings, we found that circular DNA size does not have a clear effect on cleavage rate (Supplementary Figure S2). An alternative explanation is that there is some level of *in trans* activation (e.g. see below) that is possible on the catenane due to the topological linking of the rings.

If only a single R_2_H_2_ complex were involved in DNA cleavage, then we might expect a nicked DNA intermediate to accumulate since cleavage of the second strand would require rearrangement of the complex. However, CglI leaves max. 25–30% OC and FLL starts to accumulate immediately after initiation of the reaction. While BfiI using a single active site for a double stranded break generation produces ∼80% OC and FLL starts to accumulate later (29). We propose that this is consistent with two R_2_H_2_ complexes (each containing only a single nuclease active site) being required during DNA cleavage and preceded by a rate-limiting step or series of steps, which in this case, we suggest, is a communication process (see below). The product accumulation pattern and apparent cleavage rate constants were same in the case of two-site plasmid substrates with different target orientations (HtT and TtT) (Supplementary Table S3 and Figure S3). Run-off sequencing analysis of the cleavage products showed that in all cases a double strand break is introduced at the same position near the asymmetric recognition sequence (GCCGCN_6_/N_6–7_) (Supplementary Figure S4). This indicates that the same CglI collision complex must be produced regardless of the number of targets or the relative orientation of pairs of targets.

The DNA cleavage results are not consistent with an enzyme that uses a 3D route to communicate between sites (i.e. tetrameric Type II restriction endonucleases SfiI, Cfr10I, NaeI and NgoMIV require interaction with two recognition sites before displaying their optimal DNA cleavage activities and that their interactions with two DNA sites occur through 3D space) (35,38). Although a simple interpretation of the results is that multiple sites are not required for cleavage, long-range communication via 1D process cannot be ruled out. For example, the ATP-dependent Type I RM enzymes are also able to efficiently cleave one-site plasmid, but must do so by extensive 1D translocation around the DNA circle. On a linearized version of the same DNA, cleavage does not occurs as the translocating Type I motors cannot collide. Therefore to test this possibility for CglI, we needed to explore the effect of DNA topology on cleavage activity.

We tested the relative DNA cleavage activity on one-site DNA substrates that were: negatively supercoiled circles, topologically unconstrained relaxed circles or topologically unconstrained full-length linear DNA (Figure 3A). The rate of appearance of dsDNA cleaved product of one site circular DNA was the same as on relaxed (nicked) or supercoiled DNA (Figure 3A). However, the one-site linear DNA only supported a relatively small amount of DNA cleavage (Figure 3A). This result is similar to what has been observed with Type I (and Type ISP nicking) reactions (15,39) and supports a communication mechanism along the 1D contour. Therefore we propose that the cleavage of the one-site circular DNA is activated by an enzyme moving around the whole contour of the DNA from the site and two sites are not necessary. However, on linear DNA the same communication will be largely inefficient due to the enzymes dissociating from the ends of the DNA. We propose that the small amount of cleavage of one-site linear DNA results from *in trans* interactions between CglI complexes each bound (or originating from) separate DNA molecules. Only a small fraction of DNA is captured in such complexes, therefore, small amplitude of DNA cleavage is observed. The ability of CglI to occasionally communicate *in trans* is considered below in the translocation assays.

**Figure 4.**
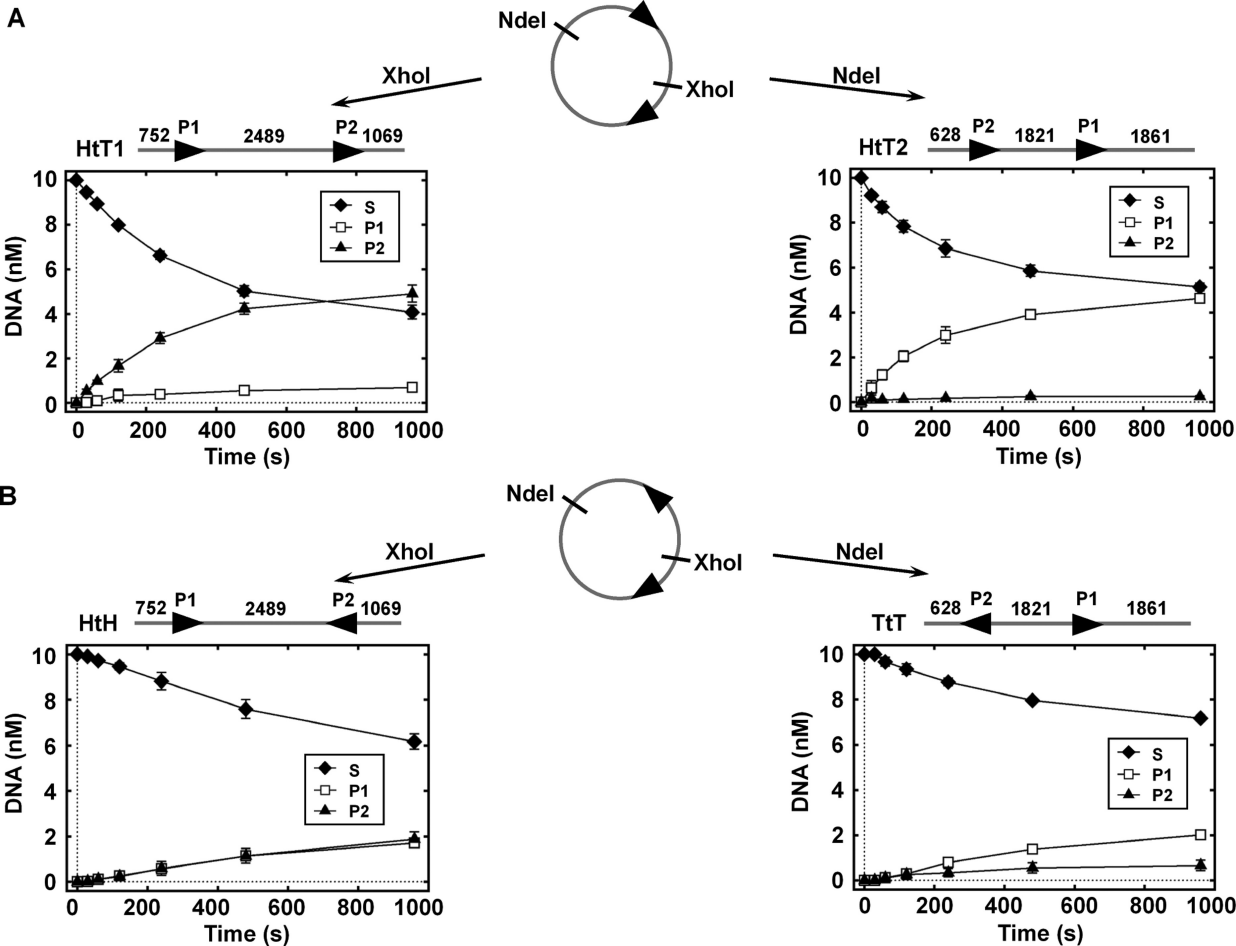
Cleavage of the two-site linear DNAs with different target orientations by CglI. Cleavage of the HtT substrates is presented in (**A**), the HtH and TtT substrates–in (**B**). The two-site supercoiled circular plasmids were used to form the linear substrates by cleavage with the restriction endonucleases XhoI and NdeI). The CglI recognition sequence (5′-GCCGC-3′) is shown as an arrowhead (▸),‘S’ represents the substrates and any nicked intermediates, while P1 and P2 represent a double strand break at site P1 or P2, respectively. Numbers indicate distances in base pair between the CglI targets and DNA ends. All reactions contained 10 nM DNA, 4 mM ATP, 500 nM R_2_H_2_·CglI and were conducted as described in ‘Materials and Methods’ section. The rate constant values (3.3 ± 0.1 × 10^−3^ s^−1^ for HtT1, 4.4 ± 0.4 × 10^−3^ s^−1^ for HtT2, 8.9 ± 1.9 × 10^−4^ s^−1^ for HtH and 2.0 ± 0.2 × 10^−3^ for TtT) were obtained by fitting a single exponential to the time courses of substrates. Points are averages with error bars as standard deviation for at least three repeat reactions.

Although cleavage of one-site linear DNA is not efficient, in the presence of multiple targets on linear DNA, cleavage may be activated by 1D interactions between enzymes initially bound at the separate sites. Moreover, a site orientation preference may be revealed using such substrates, as seen for the Type ISP and III RM enzymes (16,17,20). To test this we used four different two-site linear DNA substrates: HtT1 and HtT2—generated from two-site HtT plasmid DNA digestion with XhoI and NdeI, respectively; and, HtH and TtT: generated from two-site TtT plasmid DNA digestion with XhoI and NdeI, respectively (Figure 4).

The cleavage data show that linear DNA with a pair of sites in direct repeat (HtT1 or HtT2) is the most efficiently cut, where cleavage dominates at the second site (downstream to the first as drawn in Figure 4), with a rate that is similar to that of the one-site circular DNA and which is independent of local sequence (∼10^−3^ s^−1^, Supplementary Table S3). This would be consistent with a rear-end collision mechanism for activation of cleavage that is different from the Type I, ISP and III RM enzymes (5,16,20,22,40,41). However, we also observed cleavage of both DNA with sites in inverted repeat (HtH and TtT) with a similar rate (∼10^−4^ s^−1^) that is 10-fold slower than the direct repeats (Figure 4B and Supplementary Table S3). Therefore, although this is not a strict site-orientation preference as it is seen with Type ISP and Type III RM enzymes, there is still ∼10-fold kinetic preference for the sites in direct repeat, which also differs from the Type I RM enzymes. This points to a unique mechanism for CglI. The preference is not observed on the circular DNA as we suggest that single-site communication events occur with a faster rate on these substrates.

One explanation for an ability to cut pairs of the sites without site-orientation preference is that the communication is bidirectional, as shown for the Type I RM enzymes (10–12). A prediction of this would be that on two-site linear DNA there is a possibility to cut both sites. However, for HtT1 and HtT2, there was ∼10-fold preference for cleavage at one of the two sites (Figure 4A), while for TtT, there was ∼3-fold preference for one site of the two sites (Figure 4B). In contrast, for HtH, there was no apparent preference between the sites (Figure 4B). This data appear to support alternative DNA cleavage pathways depending on the DNA substrate. The most efficient cleavage occurs where the enzymes can communicate directionally downstream from the 5′-GCCGC-3′ site. This would produce a rear-end collision with an enzyme bound at the downstream site, where cleavage is activated (Figure 4A). Alternatively, less efficient cleavage is produced when the sites are in other orientations (Figure 4B). This alternative mechanism needs to accommodate the fact that both head-to-head and tail-to-tail arrangements can produce cleavage. This would predict some sort of bidirectional translocation or an alternative communication mechanism that is independent of the site orientation preference (such as reversal of directionality of communication).

### Evidence for bidirectional translocation of CglI on DNA

**Figure 5.**
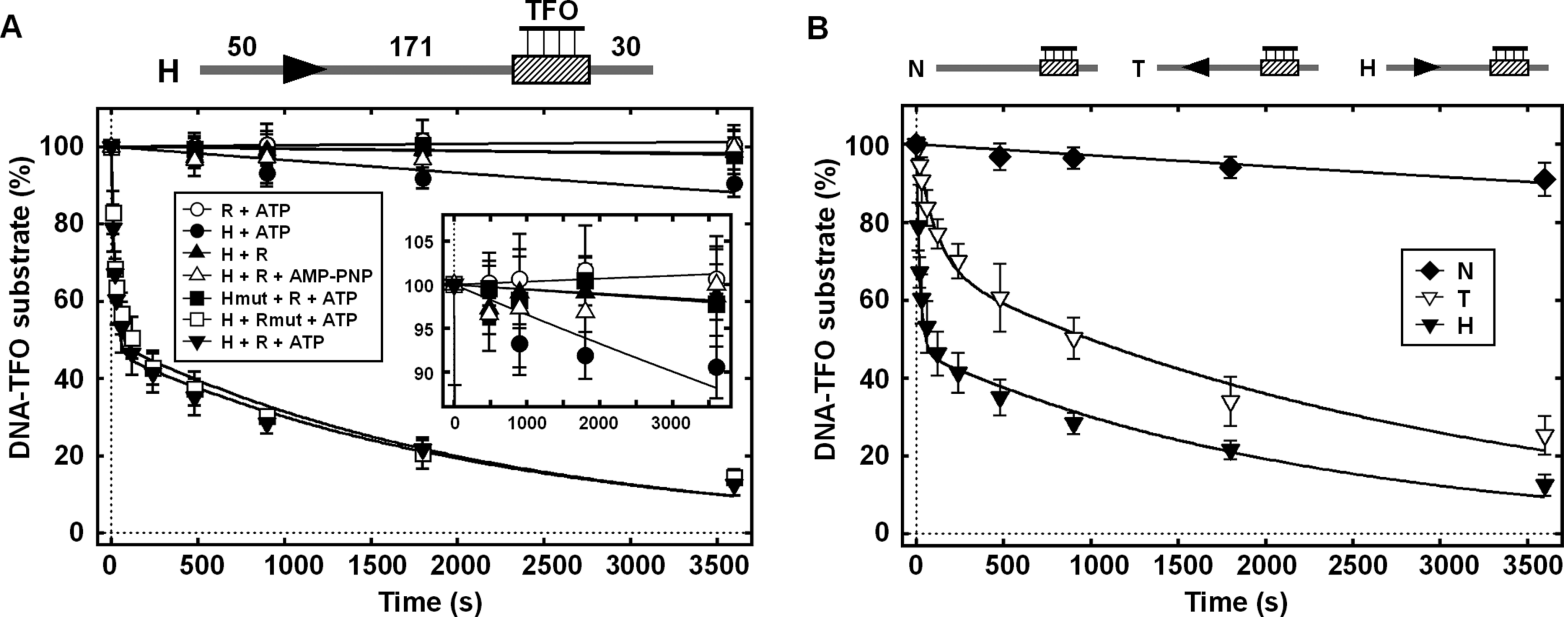
CglI translocase activity analyzed using the DNA triplex displacement assay. (**A**) CglI displaces TFO. Scheme of the DNA substrate used in the triplex assay is shown above the graph: DNA is shown as a thick line, the CglI recognition sequence (5′-GCCGC-3′) is shown as an arrowhead (▸), the triplex binding sequence is shown as a varied rectangle, numbers indicate distances in base pair. Used abbreviations: H—H.CglI, R—R.CglI, Hmut—the H.CglI (D158A + E159A) mutant, Rmut—the R.CglI (H105A) mutant. The *inset* shows zoomed part of the graph to clarify the differences of TFO displacement. (**B**) TFO displacement by CglI is dependent on the recognition sequence. DNA substrates: N—DNA without the recognition sequence, the T and H DNA fragments contain the recognition sequence in both orientations. All reactions contained 5 nM DNA, 2.5 nM TFO, 4 mM ATP, 200 nM R.CglI, 200 nM H.CglI and were conducted as described in ‘Materials and Methods’ section. Solid lines are single or double exponential fits to the data. Rate constants for TFO displacement are presented in Supplementary Table S4. Points are averages with error bars as standard deviation for at least three repeat reactions.

Previously it was shown that H.CglI carries SF2 helicase motifs (28). These motifs are also found in Type I and III RM enzymes and are required for ATP hydrolysis-driven DNA translocation or activation of DNA sliding (19,28). The rapid DNA cleavage seen above with one site plasmids or with linear DNA with two sites in direct repeat supports a long-range communication mechanism that follows the 1D DNA contour, principally, but not exclusively, downstream of the 5′-GCCGC-3′ site. Given the relatively high level of ATP hydrolysis by CglI (28), we would suggest that this communication is due to stepwise DNA translocation rather than sliding. To test this, we used the triplex displacement assay, which was successfully applied for Type I and ISP RM enzymes (42,43), and other dsDNA translocating motors (44–46). When located downstream of the 5′-GCCGC-3′ site, triplex displacement was observed dependent upon ATP hydrolysis and a WT helicase domain (Figure 5A). Mutation of the nuclease motif (R_mut_) had no effect. Triplex displacement is consistent with a translocating motor. There were two distinct phases to the triplex displacement profile; a first phase of ∼50% amplitude with a rate ∼10^−2^ s^−1^ and a second phase of ∼50% amplitude with a rate ∼10^−4^ s^−1^ (Figure 5A and Supplementary Table S4).

To confirm the directionality of communication, we tested non-specific DNA without a CglI site or specific DNA where the triplex was located upstream of the target (Figure 5B). On the non-specific DNA we observed a markedly slower rate of displacement, which most likely reflects non-specific displacement due to protein-dependent and -independent effects that are not related to translocation *per se* (Figure 5A). When the triplex was formed upstream the recognition site, we also observed a biphasic displacement profile, with a first phase of ∼30% amplitude and a rate ∼10^−3^ s^−1^, and with a second phase of ∼70% amplitude and a rate ∼10^−4^ s^−1^ (Figure 5B and Supplementary Table S4). As observed with the cleavage data, the triplex displacement data appear to support two different communication mechanisms with differing efficiencies. Rapid triplex displacement is due to translocation *downstream* of the site while slower triplex displacement is due to translocation *upstream* of the site. An even slower second phase of displacement was seen on both DNA with a similar rate. We note that we did not observe a clear lag phase characteristic of a stepping motor (47). However, similar profiles have been observed with other *bona fide* translocases such as Mfd (48), where the absence of a clear lag may be due to events prior to translocation (DNA binding, initiation) being rate-limiting. The biphasic nature of the displacement profiles may reflect dissociation of a subset of translocating enzymes that then reinitiate (9). We also tested translocation using a protein (*Streptococcus thermophilus* CRISPR Cas9 or Cascade) displacement assay (Supplementary Figure S5). This data also support communication both up- and downstream but with a preference for downstream translocation.

### Formation of protein–DNA sandwich complexes allows translocation activity *in trans*

One explanation for the above data is that CglI can translocate bidirectionally but that the efficiency and translocation rate is different in the downstream and upstream directions. An alternative hypothesis is that CglI can only translocate unidirectionally, but that the motor can switch directions with a slower rate. This directional switch may be due to the enzyme leaving the DNA completely, remaining activated in free solution and then rebinding a new DNA site, either on the same DNA molecule or on a different DNA molecule. This rebinding would be randomized so that subsequent translocation would no longer be dictated by the site orientation. Alternatively, since the R_2_H_2_ complex has two DNA binding and two motor domains, there could be a simultaneous binding of a second DNA location without dissociation from the first. This could result in both DNA loops (binding of two sites on the same DNA) and formation of DNA sandwiches (binding of two sites, each on different DNA). Both types of event would randomize the direction of subsequent translocation.

**Figure 6.**
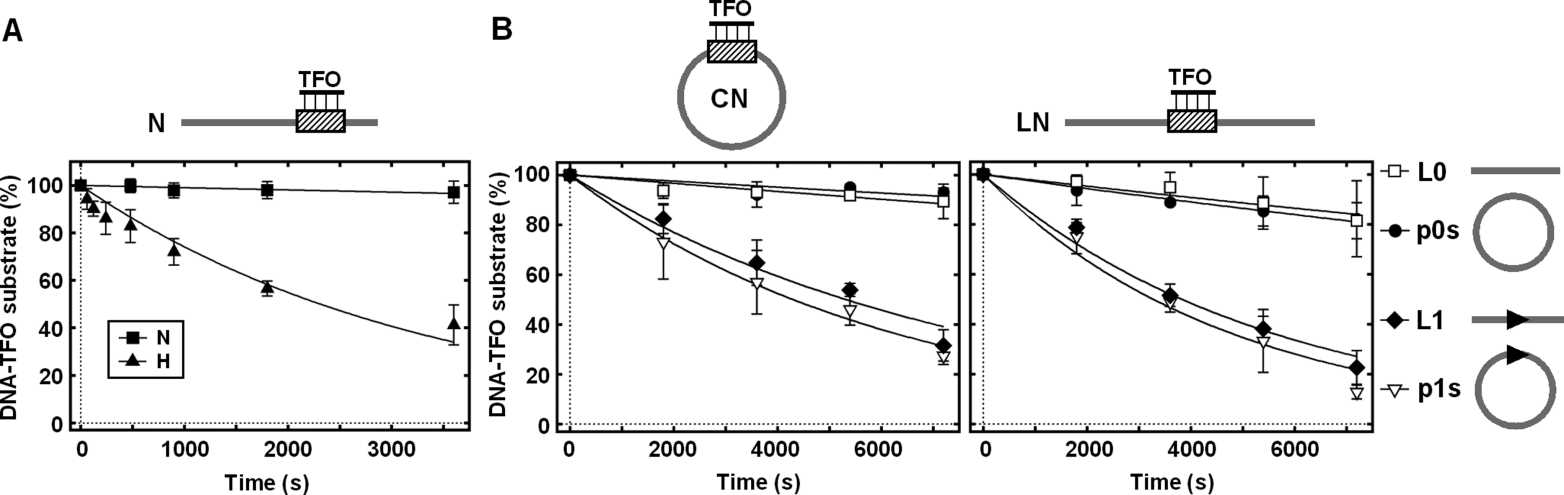
TFO displacement by CglI *in trans*. (**A**) TFO displacement from the linear DNA (N) without the recognition sequence of CglI. DNA substrate (278 bp in length) is shown above the graph. The triplex binding sequence is shown as a varied rectangle. DNA added *in trans* (278 bp in length) are the same as used in Figure 5B. (**B**) TFO displacement from the circular versus linear DNA. DNA substrates (3383 bp in length) are shown above graph. The CglI recognition sequence (5′-GCCGC-3′) is shown as an arrowhead (▸). DNA added *in trans* (3105 bp in length) were linear (L0 and L1) and circular (p0s and p1s) without and with recognition sequence of CglI (are shown on the right). All reactions contained 1 nM DNA substrate, 0.5 nM TFO, 4 mM ATP, 200 nM R.CglI (H105A), 200 nM H.CglI, 4 nM (A) or 10 nM (B) DNA added *in trans* and were conducted as described in ‘Materials and Methods’ section. Solid lines are single exponential fits to the data. Rate constants are presented in Supplementary Table S4. Points are averages with error bars as standard deviation for at least three repeat reactions.

First, we tested whether motor activity can transfer from one DNA molecule to another. In the absence of a CglI target site on a linear DNA, triplex displacement was not observed above background levels (Figure 5B). We then mixed this DNA with a second linear DNA molecule either without a target (‘N’) or with a target (‘H’) (Figure 6A). Triplex displacement was observed when a CglI site was added *in trans*, suggesting that the motor activity can switch between DNA molecules, with the second binding event occurring on non-specific DNA. We repeated this experiment using different combinations of circular or linear triplex DNA with circular or linear specific, or with circular or linear non-specific DNA (Figure 6B). Activation of triplex displacement was observed in all cases where a CglI site was provided *in trans*. Therefore, the transfer between DNA molecules does not require a DNA end, either for CglI exit from the specific DNA or for entry on to the non-specific DNA (Figure 6B).

**Figure 7.**
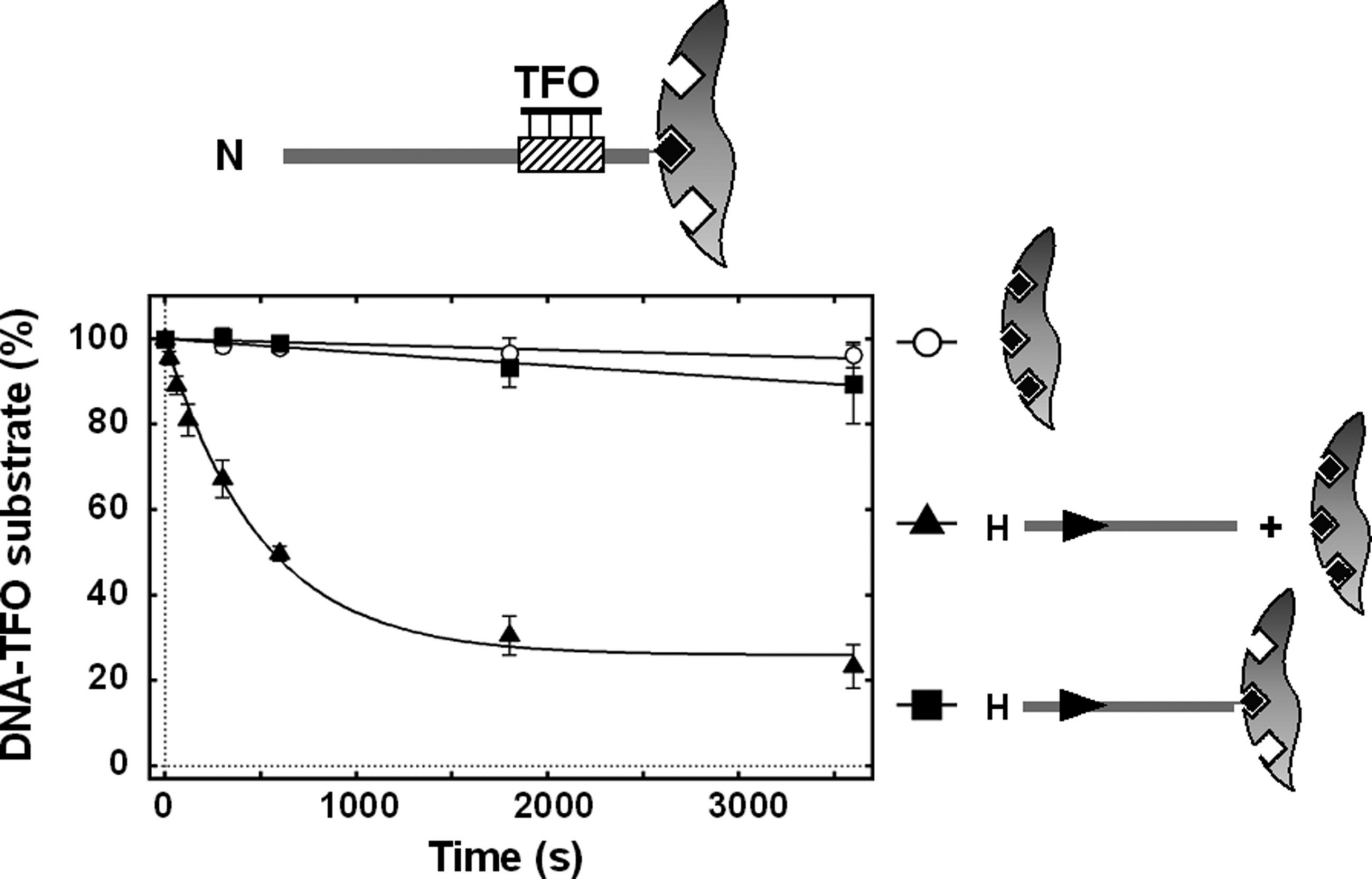
Effect of DNA immobilization on TFO displacement by CglI *in trans*. TFO displacement from the non-specific DNA in the presence of the specific DNA (in solution or immobilized). N, H and T DNA fragments (278 bp in length) are the same as used in Figure 5B. DNA is shown as a thick line, the CglI recognition sequence (5′-GCCGC-3′) is shown as an arrowhead (▸), the triplex binding sequence is shown as a varied rectangle, biotin is shown as black diamond, magnetic bead is shown as gray circle. All reactions contained 1 nM DNA substrate, 0.5 nM TFO, 4 mM ATP, 200 nM R.CglI, 200 nM H.CglI, 50 nM DNA *in trans* (in solution or immobilized) and were conducted as described in ‘Materials and Methods’ section. Solid lines are single exponential fits to the data. Rate constants are presented in Supplementary Table S4. Points are averages with error bars as standard deviation for at least three repeat reactions.

To distinguish between single motor (dissociation/association) and double motor (looping/sandwich) transfer mechanisms, we measured triplex displacement activity using DNA bound to magnetic beads. Where DNA molecules are bound to separate beads, exclusion effects prevent the formation of sandwich complexes between those DNA (49,50). Therefore any transfer must occur via free solution due to dissociation–association events. We tested this by mixing specific or non-specific biotinylated linear triplex DNA, either bound or not bound to streptavidin-coated magnetic beads, with a specific biotinylated linear DNA either bound or not bound to streptavidin coated magnetic beads. The results in Figure 7 indicate that transfer cannot occur via free solution. Also we tried to catch the activated CglI translocase by removing the biotinylated activator DNA (contains the CglI recognition sequence) using streptavidin coated magnetic beads and then adding the non-specific DNA triplex, however, no TFO displacement was observed. When non-specific triplex DNA was immobilized on the magnetic beads and mixed with magnetic beads bearing the immobilized specific DNA to prevent simultaneous CglI binding with both specific (activator) and non-specific DNA, triplex displacement was not observed (Figure 7). In contrast, the triplex was displaced when non-immobilized specific DNA was added in solution (Figure 7). This indicates that CglI requires simultaneous binding both specific and non-specific DNA for the TFO displacement *in trans*.

Under equivalent experimental conditions, the rate of triplex displacement (∼10^−4^ s^−1^) due to *in trans* transfer (Figure 6A) was of the same order as the rates of the second phases of triplex displacement observed in Figure 5, (Supplementary Table S4). Therefore, it is possible that at least some of the observed triplex displacement activity in Figure 5 is due to *in trans* transfers of translocation activity between DNA molecules. Such events would lose the original site orientation. When the triplex was located upstream of the CglI site, the initial triplex displacement rate was faster than the *in trans* transfer (Figure 5B). This could be due to (i) a slower upstream translocation mechanism that is still more efficient that *in trans* transfer; and/or, (ii) a more efficient *in cis* transfer due to DNA looping. For the Type III RM enzymes, a protein roadblock assay was used to rule out looping activities (21). However, the vigorous protein displacement activity of the translocating CglI means that we cannot repeat a similar assay to prove or disprove looping of the motor.

As noted above, the inefficient cleavage of the one-site linear DNA (Figure 3) may be due to inefficient *in trans* transfer events. In principle the *in trans* transfer can be improved by increasing the local concentration of the protein–DNA complex relative to the second DNA. To test this, we measured the cleavage of a one-site linear DNA in the presence of a fixed concentration of CglI and increasing concentrations of a second, smaller one-site DNA (Supplementary Figure S6). An increased efficiency of cleavage was observed as concentration of the second DNA increased, consistent with *in trans* transfer playing a role in cleavage where direct 1D communication is not possible.

Why would an RM enzyme show site orientation preference during long-range communication? For the Type ISP and III enzymes, a simple explanation is that the accompanying methyltransferase activity only modifies one strand (17,51,52). This hemimethylation pattern would result in unmodified sites following semi-conservative replication. However, by requiring pairs of sites in inverted repeat orientation for cleavage, any unmodified site is only aligned with modified sites and cleavage is prevented, allowing maintenance methylation. We currently do not know the methylation pattern for the CglI-like enzymes. However, we note that unlike the Type ISP and III enzymes, there is a less clear-cut site orientation preference for CglI and the results may be more consistent with methylation of both strands which accommodates bidirectional translocation, as for Type I RM enzymes (10).

## CONCLUSION

**Figure 8.**
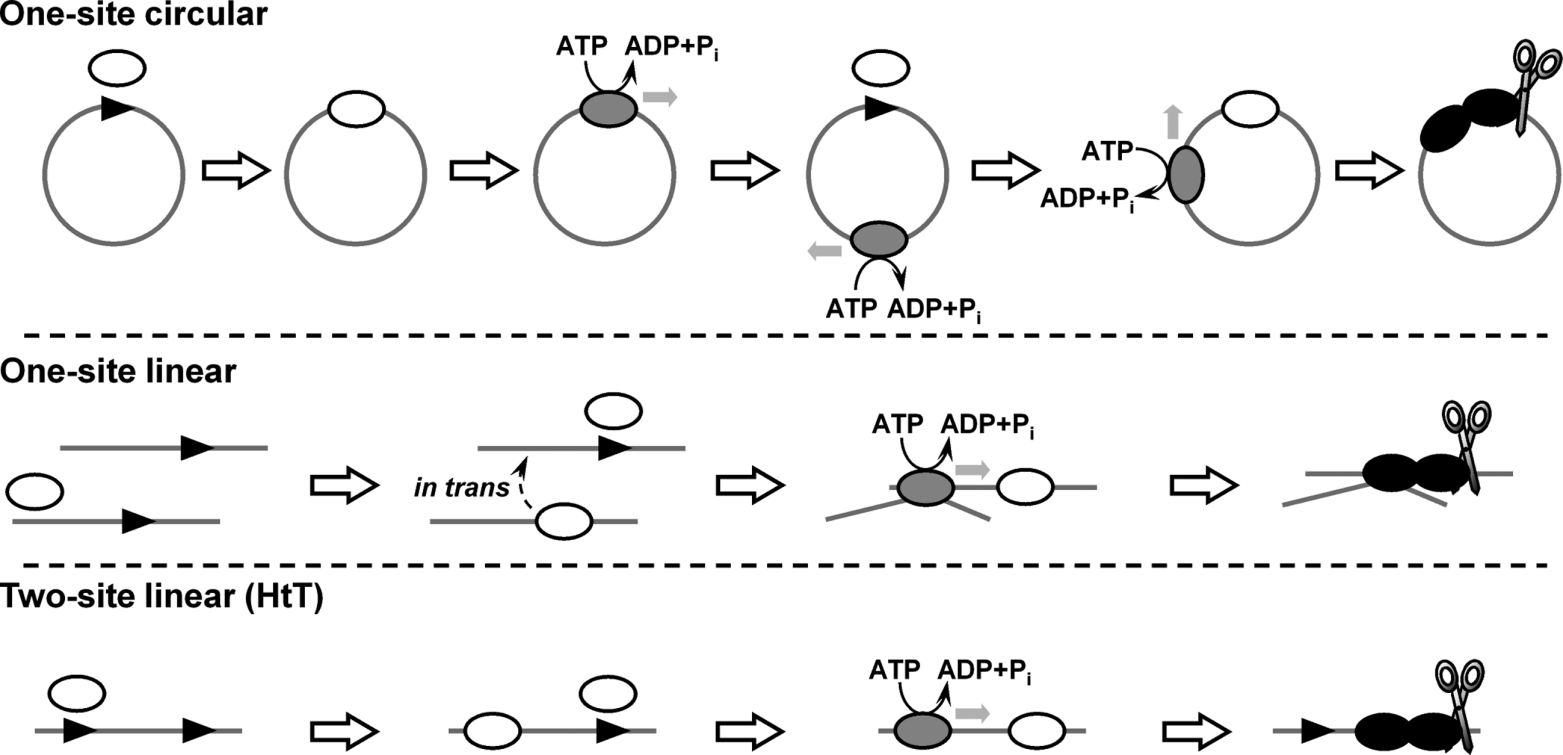
Proposed mechanism of action of CglI on various DNA substrates. DNA is shown as a thick line, the CglI recognition sequence (5′-GCCGC-3′) is shown as an arrowhead (▸). The R_2_H_2_·CglI complex (white ellipse) containing only a single active site binds the recognition sequence, becomes activated (gray ellipse), starts to translocate on DNA using ATP hydrolysis and leaves the target. For clarity here only one of two possible directions of translocation on DNA is shown. The second CglI complex binds to the same recognition sequence. After collision of the target-bound and translocating CglI complexes (black ellipses) a double-stranded break is introduced 7 and 6/7 nucleotides downstream of the 5′-GCCGC-3′ site on the top and bottom strands, respectively. In the case of the linear one-site substrate two target bound/activated CglI complexes associate *in trans* introducing a double-stranded break near to the one of the targets (inefficient cleavage). For the linear two-site substrates only a scheme of the HtT fragment cleavage is shown. CglI shows a preferential directionality of translocation on DNA (shown by an arrow) resulting in the predominant DNA cleavage at the second site (downstream to the first).

On the basis of the data presented in this article, we make a number of conclusions and propose alternative cleavage mechanisms to account for the CglI cleavage activity on the different DNA substrates (Figure 8):

- A single site plasmid DNA is sufficient for efficient DNA cleavage performed by two CglI complexes. This DNA cleavage requires long-range communication by one of the complexes *in cis* along the 1D contour.
- Communication is most likely driven by a stepwise ATP hydrolysis-dependent translocation mechanism that is active both downstream ad upstream of the site (i.e. bidirectionally with some preference downstream of the asymmetric 5′-GCCGC-3′ site). The enzyme leaves the site and the original binding orientation of the protein is retained. Motor activity can even produce sufficient force to displace stable R-loop structures of CRISPR effector complexes.
- Unusually, this translocation activity can transfer onto a new DNA substrate with low efficiency. The transfer between DNA molecules does not require a DNA end, either for CglI exit from the specific DNA or for binding to the non-specific DNA, and results from simultaneous interaction between both the DNAs.
- The most efficient cleavage activity is due to rear-end collision between a target-bound complex and a translocating complex. Consequently, circular DNA and linear DNA with sites in direct repeat (i.e. HtT) DNA are the best cleavage substrates.
- Cleavage can occur with lower efficiency on linear with a single site or pairs of sites in inverted repeat (either HtH or TtT). This is due to a combination of upstream translocation events and/or motor transfers events *in cis* (looping) or *in trans* (sandwich complexes).
- Regardless of the pathway to activation of the nuclease activity, cleavage always occurs at the same location (GCCGCN_6_/N_6–7_). This indicates that the same CglI collision complex must be produced by different pathways of communication.
- Data from a related enzyme, BceSIV, has been interpreted to show cleavage on both sides of the pseudo-palindromic site GCWGC (53). Although we do not believe that our data support cleavage on both sides of the site, there is the possibility that CglI can occasionally load onto the site in a GCGGC orientation and initiate translocation ‘upstream’. This would allow bi-directional translocation but with a strong bias in one direction. Although this is a possibility, the difference in the rates of cleavage of HtH and TtT substrates (Figure 4B) would suggest that there should be more cleavage upstream of the site than observed.

Based on these points, the CglI enzyme does not easily conform to classification as a Type I, ISP or III RM enzyme. Since stepwise translocation is the more likely mode of communication, the closest resemblance is to the Type I enzymes. Single molecule experiments are underway to confirm or refute the bidirectional DNA translocation mechanism of CglI enzyme during the cleavage of DNA.

## Supplementary Material

Supplementary Data

## References

[1] Loenen W.A., Dryden D.T., Raleigh E.A., Wilson G.G., Murray N.E. Highlights of the DNA cutters: a short history of the restriction enzymes. Nucleic Acids Res. 2014; 42:3–19. 24141096 10.1093/nar/gkt990 PMC3874209

[2] Loenen W.A., Dryden D.T., Raleigh E.A., Wilson G.G. Type I restriction enzymes and their relatives. Nucleic Acids Res. 2014; 42:20–44. 24068554 10.1093/nar/gkt847 PMC3874165

[3] Rao D.N., Dryden D.T., Bheemanaik S. Type III restriction-modification enzymes: a historical perspective. Nucleic Acids Res. 2014; 42:45–55. 23863841 10.1093/nar/gkt616 PMC3874151

[4] Pingoud A., Jeltsch A. Structure and function of type II restriction endonucleases. Nucleic Acids Res. 2001; 29:3705–3727. 11557805 10.1093/nar/29.18.3705 PMC55916

[5] Szczelkun M.D., Friedhoff P., Seidel R. Maintaining a sense of direction during long-range communication on DNA. Biochem. Soc. Trans. 2010; 38:404–409. 20298192 10.1042/BST0380404 PMC2860699

[6] Szczelkun M.D. Translocation, switching and gating: potential roles for ATP in long-range communication on DNA by Type III restriction endonucleases. Biochem. Soc. Trans. 2011; 39:589–594. 21428945 10.1042/BST0390589 PMC3064402

[7] Peakman L.J., Szczelkun M.D. DNA communications by Type III restriction endonucleases–confirmation of 1D translocation over 3D looping. Nucleic Acids Res. 2004; 32:4166–4174. 15302916 10.1093/nar/gkh762 PMC514383

[8] Ramanathan S.P., van Aelst K., Sears A., Peakman L.J., Diffin F.M., Szczelkun M.D., Seidel R. Type III restriction enzymes communicate in 1D without looping between their target sites. Proc. Natl. Acad. Sci. U.S.A. 2009; 106:1748–1753. 19181848 10.1073/pnas.0807193106 PMC2633214

[9] Chand M.K., Nirwan N., Diffin F.M., van Aelst K., Kulkarni M., Pernstich C., Szczelkun M.D., Saikrishnan K. Translocation-coupled DNA cleavage by the Type ISP restriction-modification enzymes. Nat. Chem. Biol. 2015; 11:870–877. 26389736 10.1038/nchembio.1926 PMC4636054

[10] Firman K., Szczelkun M.D. Measuring motion on DNA by the type I restriction endonuclease EcoR124I using triplex displacement. EMBO J. 2000; 19:2094–2102. 10790375 10.1093/emboj/19.9.2094 PMC305691

[11] Bianco P.R., Hurley E.M. The type I restriction endonuclease EcoR124I, couples ATP hydrolysis to bidirectional DNA translocation. J. Mol. Biol. 2005; 352:837–859. 16126220 10.1016/j.jmb.2005.07.055

[12] Bianco P.R., Xu C., Chi M. Type I restriction endonucleases are true catalytic enzymes. Nucleic Acids Res. 2009; 37:3377–3390. 19336412 10.1093/nar/gkp195 PMC2691833

[13] Seidel R., Bloom J.G., Dekker C., Szczelkun M.D. Motor step size and ATP coupling efficiency of the dsDNA translocase EcoR124I. EMBO J. 2008; 27:1388–1398. 18388857 10.1038/emboj.2008.69 PMC2291450

[14] Smith R.M., Josephsen J., Szczelkun M.D. The single polypeptide restriction-modification enzyme LlaGI is a self-contained molecular motor that translocates DNA loops. Nucleic Acids Res. 2009; 37:7219–7230. 19783815 10.1093/nar/gkp794 PMC2790907

[15] van Aelst K., Sisakova E., Szczelkun M.D. DNA cleavage by type ISP restriction-modification enzymes is initially targeted to the 3′-5′ strand. Nucleic Acids Res. 2013; 41:1081–1090. 23221632 10.1093/nar/gks1210 PMC3553963

[16] Sisakova E., van Aelst K., Diffin F.M., Szczelkun M.D. The type ISP restriction-modification enzymes LlaBIII and LlaGI use a translocation-collision mechanism to cleave non-specific DNA distant from their recognition sites. Nucleic Acids Res. 2013; 41:1071–1080. 23222132 10.1093/nar/gks1209 PMC3553950

[17] Smith R.M., Diffin F.M., Savery N.J., Josephsen J., Szczelkun M.D. DNA cleavage and methylation specificity of the single polypeptide restriction-modification enzyme LlaGI. Nucleic Acids Res. 2009; 37:7206–7218. 19808936 10.1093/nar/gkp790 PMC2790903

[18] Schwarz F.W., Toth J., van Aelst K., Cui G., Clausing S., Szczelkun M.D., Seidel R. The helicase-like domains of type III restriction enzymes trigger long-range diffusion along DNA. Science. 2013; 340:353–356. 23599494 10.1126/science.1231122 PMC3646237

[19] Szczelkun M.D. Roles for helicases as ATP-dependent molecular switches. Adv. Exp. Med. Biol. 2013; 767:225–244. 23161014 10.1007/978-1-4614-5037-5_11

[20] van Aelst K., Toth J., Ramanathan S.P., Schwarz F.W., Seidel R., Szczelkun M.D. Type III restriction enzymes cleave DNA by long-range interaction between sites in both head-to-head and tail-to-tail inverted repeat. Proc. Natl. Acad. Sci. U.S.A. 2010; 107:9123–9128. 20435912 10.1073/pnas.1001637107 PMC2889075

[21] Toth J., van Aelst K., Salmons H., Szczelkun M.D. Dissociation from DNA of type III restriction-modification enzymes during helicase-dependent motion and following endonuclease activity. Nucleic Acids Res. 2012; 40:6752–6764. 22523084 10.1093/nar/gks328 PMC3413136

[22] Schwarz F.W., van Aelst K., Toth J., Seidel R., Szczelkun M.D. DNA cleavage site selection by Type III restriction enzymes provides evidence for head-on protein collisions following 1D bidirectional motion. Nucleic Acids Res. 2011; 39:8042–8051. 21724613 10.1093/nar/gkr502 PMC3185417

[23] Labrie S.J., Samson J.E., Moineau S. Bacteriophage resistance mechanisms. Nat. Rev. Microbiol. 2010; 8:317–327. 20348932 10.1038/nrmicro2315

[24] Pingoud A., Jeltsch A. Recognition and cleavage of DNA by type-II restriction endonucleases. Eur. J. Biochem. 1997; 246:1–22. 9210460 10.1111/j.1432-1033.1997.t01-6-00001.x

[25] Klimasauskas S., Kumar S., Roberts R.J., Cheng X. HhaI methyltransferase flips its target base out of the DNA helix. Cell. 1994; 76:357–369. 8293469 10.1016/0092-8674(94)90342-5

[26] Taylor J.D., Badcoe I.G., Clarke A.R., Halford S.E. EcoRV restriction endonuclease binds all DNA sequences with equal affinity. Biochemistry. 1991; 30:8743–8753. 1909572 10.1021/bi00100a005

[27] Narlikar G.J., Sundaramoorthy R., Owen-Hughes T. Mechanisms and functions of ATP-dependent chromatin-remodeling enzymes. Cell. 2013; 154:490–503. 23911317 10.1016/j.cell.2013.07.011 PMC3781322

[28] Zaremba M., Toliusis P., Grigaitis R., Manakova E., Silanskas A., Tamulaitiene G., Szczelkun M.D., Siksnys V. DNA cleavage by CgII and NgoAVII requires interaction between N- and R-proteins and extensive nucleotide hydrolysis. Nucleic Acids Res. 2014; 42:13887–13896. 25429977 10.1093/nar/gku1236 PMC4267653

[29] Sasnauskas G., Halford S.E., Siksnys V. How the BfiI restriction enzyme uses one active site to cut two DNA strands. Proc. Natl. Acad. Sci. U.S.A. 2003; 100:6410–6415. 12750473 10.1073/pnas.1131003100 PMC164460

[30] Yoshioka K. KyPlot—a user-oriented tool for statistical data analysis and visualization. Comput. Stat. 2002; 17:425–437.

[31] Simons M., Szczelkun M.D. Recycling of protein subunits during DNA translocation and cleavage by Type I restriction-modification enzymes. Nucleic Acids Res. 2011; 39:7656–7666. 21712244 10.1093/nar/gkr479 PMC3177213

[32] Roberts G.A., Cooper L.P., White J.H., Su T.J., Zipprich J.T., Geary P., Kennedy C., Dryden D.T. An investigation of the structural requirements for ATP hydrolysis and DNA cleavage by the EcoKI Type I DNA restriction and modification enzyme. Nucleic Acids Res. 2011; 39:7667–7676. 21685455 10.1093/nar/gkr480 PMC3177214

[33] Peakman L.J., Antognozzi M., Bickle T.A., Janscak P., Szczelkun M.D. S-adenosyl methionine prevents promiscuous DNA cleavage by the EcoP1I type III restriction enzyme. J. Mol. Biol. 2003; 333:321–335. 14529619 10.1016/j.jmb.2003.08.042

[34] Wood K.M., Daniels L.E., Halford S.E. Long-range communications between DNA sites by the dimeric restriction endonuclease SgrAI. J. Mol. Biol. 2005; 350:240–253. 15923010 10.1016/j.jmb.2005.04.053

[35] Embleton M.L., Siksnys V., Halford S.E. DNA cleavage reactions by type II restriction enzymes that require two copies of their recognition sites. J. Mol. Biol. 2001; 311:503–514. 11493004 10.1006/jmbi.2001.4892

[36] Szczelkun M.D., Dillingham M.S., Janscak P., Firman K., Halford S.E. Repercussions of DNA tracking by the type IC restriction endonuclease EcoR124I on linear, circular and catenated substrates. EMBO J. 1996; 15:6335–6347. 8947056 PMC452456

[37] Marshall J.J., Gowers D.M., Halford S.E. Restriction endonucleases that bridge and excise two recognition sites from DNA. J. Mol. Biol. 2007; 367:419–431. 17266985 10.1016/j.jmb.2006.12.070 PMC1892151

[38] Szczelkun M.D., Halford S.E. Recombination by resolvase to analyse DNA communications by the SfiI restriction endonuclease. EMBO J. 1996; 15:1460–1469. 8635479 PMC450051

[39] Szczelkun M.D., Janscak P., Firman K., Halford S.E. Selection of non-specific DNA cleavage sites by the type IC restriction endonuclease EcoR124I. J. Mol. Biol. 1997; 271:112–123. 9300058 10.1006/jmbi.1997.1172

[40] van Aelst K., Saikrishnan K., Szczelkun M.D. Mapping DNA cleavage by the Type ISP restriction-modification enzymes following long-range communication between DNA sites in different orientations. Nucleic Acids Res. 2015; 43:10430–10443. 26507855 10.1093/nar/gkv1129 PMC4666363

[41] Kulkarni M., Nirwan N., van Aelst K., Szczelkun M.D., Saikrishnan K. Structural insights into DNA sequence recognition by type ISP restriction-modification enzymes. Nucleic Acids Res. 2016; 44:4396–4408. 26975655 10.1093/nar/gkw154 PMC4872093

[42] Seidel R., Bloom J.G., van Noort J., Dutta C.F., Dekker N.H., Firman K., Szczelkun M.D., Dekker C. Dynamics of initiation, termination and reinitiation of DNA translocation by the motor protein EcoR124I. EMBO J. 2005; 24:4188–4197. 16292342 10.1038/sj.emboj.7600881 PMC1356320

[43] Stanley L.K., Seidel R., van der Scheer C., Dekker N.H., Szczelkun M.D., Dekker C. When a helicase is not a helicase: dsDNA tracking by the motor protein EcoR124I. EMBO J. 2006; 25:2230–2239. 16642041 10.1038/sj.emboj.7601104 PMC1462981

[44] Blackwood J.K., Rzechorzek N.J., Abrams A.S., Maman J.D., Pellegrini L., Robinson N.P. Structural and functional insights into DNA-end processing by the archaeal HerA helicase-NurA nuclease complex. Nucleic Acids Res. 2012; 40:3183–3196. 22135300 10.1093/nar/gkr1157 PMC3326311

[45] Graham J.E., Sherratt D.J., Szczelkun M.D. Sequence-specific assembly of FtsK hexamers establishes directional translocation on DNA. Proc. Natl. Acad. Sci. U.S.A. 2010; 107:20263–20268. 21048089 10.1073/pnas.1007518107 PMC2996697

[46] Smith A.J., Szczelkun M.D., Savery N.J. Controlling the motor activity of a transcription-repair coupling factor: autoinhibition and the role of RNA polymerase. Nucleic Acids Res. 2007; 35:1802–1811. 17329375 10.1093/nar/gkm019 PMC1874598

[47] McClelland S.E., Dryden D.T., Szczelkun M.D. Continuous assays for DNA translocation using fluorescent triplex dissociation: application to type I restriction endonucleases. J. Mol. Biol. 2005; 348:895–915. 15843021 10.1016/j.jmb.2005.03.018

[48] Smith A.J., Pernstich C., Savery N.J. Multipartite control of the DNA translocase, Mfd. Nucleic Acids Res. 2012; 40:10408–10416. 22904071 10.1093/nar/gks775 PMC3488230

[49] Zaremba M., Owsicka A., Tamulaitis G., Sasnauskas G., Shlyakhtenko L.S., Lushnikov A.Y., Lyubchenko Y.L., Laurens N., van den Broek B., Wuite G.J. DNA synapsis through transient tetramerization triggers cleavage by Ecl18kI restriction enzyme. Nucleic Acids Res. 2010; 38:7142–7154. 20571089 10.1093/nar/gkq560 PMC2978343

[50] Gasiunas G., Sasnauskas G., Tamulaitis G., Urbanke C., Razaniene D., Siksnys V. Tetrameric restriction enzymes: expansion to the GIY-YIG nuclease family. Nucleic Acids Res. 2008; 36:938–949. 18086711 10.1093/nar/gkm1090 PMC2241918

[51] Meisel A., Mackeldanz P., Bickle T.A., Kruger D.H., Schroeder C. Type III restriction endonucleases translocate DNA in a reaction driven by recognition site-specific ATP hydrolysis. EMBO J. 1995; 14:2958–2966. 7796821 10.1002/j.1460-2075.1995.tb07296.x PMC398416

[52] Meisel A., Bickle T.A., Kruger D.H., Schroeder C. Type III restriction enzymes need two inversely oriented recognition sites for DNA cleavage. Nature. 1992; 355:467–469. 1734285 10.1038/355467a0

[53] Xu S.Y., Nugent R.L., Kasamkattil J., Fomenkov A., Gupta Y., Aggarwal A., Wang X., Li Z., Zheng Y., Morgan R. Characterization of type II and III restriction-modification systems from Bacillus cereus strains ATCC 10987 and ATCC 14579. J. Bacteriol. 2012; 194:49–60. 22037402 10.1128/JB.06248-11 PMC3256598

[54] Endlich B., Linn S. The DNA restriction endonuclease of Escherichia coli B. II. Further studies of the structure of DNA intermediates and products. J. Biol. Chem. 1985; 260:5729–5738. 2985610

[55] Kimball M., Linn S. The release of oligonucleotides by the Escherichia coli B restriction endonuclease. Biochem. Biophys. Res. Commun. 1976; 68:585–591. 766758 10.1016/0006-291x(76)91185-2

