# Supplementary material for: CgII cleaves DNA using a mechanism distinct from other ATP-dependent restriction endonucleases
Source: Nucleic Acids Res. 2017 Jul 7;45(14):8435–47. doi: 10.1093/nar/gkx580 (PMC5737866; doi:10.1093/nar/gkx580)
Supplement: Supplementary Data [file gkx580_supp.pdf]

# Supplementary data to:

# CgII cleaves DNA using a mechanism distinct from other ATP-dependent restriction endonucleases

Paulius Toliusis[1], Mindaugas Zaremba[1,*], Arunas Silanskas[1], Mark D. Szczelkun[2] and Virginijus Siksnys[1,*]

[1] Department of Protein–DNA Interactions, Institute of Biotechnology, Vilnius University, Sauletekio al. 7, LT-10257, Vilnius, Lithuania.
[2] DNA–Protein Interactions Unit, School of Biochemistry, Biomedical Sciences Building, University of Bristol, Bristol, BS8 1TD, UK

* To whom correspondence should be addressed. Tel: +370-5-2234357; Fax: +370-5-2234367; Email: zare@ibt.lt. Correspondence may also be addressed to Virginijus Siksnys. Tel: +370-5-2234359; Fax: +370-5-2234367; Email: siksnys@ibt.lt.

The authors wish it to be known that, in their opinion, the first two authors should be regarded as joint First Authors.

## SUPPLEMENTARY DATA

*Supplementary text*

**DNA manipulations for Cas9 and Cascade RNP (ribonucleoprotein) complex displacement**

Five different 647 bp (for Cas9) or 680 bp (for Cascade) substrates NN, HN, NT, TN, HT were synthesized by PCR using MZ-525 and MZ-528, MZ-524 and MZ-528, MZ-525 and MZ-526, MZ-523 and MZ-528, MZ-524 and MZ-526 oligonucleotides as primers and pUC-sp3 (for Cas9) or pUC19-TS132/133 (for Cascade) plasmids as templates, respectively (see Supplementary Table S1 and S2) All PCR fragments were purified using the GeneJET$^{TM}$ PCR Purification Kit (Thermo Fisher Scientific, Vilnius, Lithuania) and $^{33}$P-labelled.

**Assembly of the Cas9 RNP complex**

A ternary Cas9-crRNA-tracrRNA complex was assembled by mixing 1 µM of $(His)_6$-tagged Cas9 double mutant D10A+H840A with a pre-annealed 42 nt crRNA and 78 nt tracrRNA duplex at 1:1 molar ratio and incubated in the SB buffer (10 mM Tris-HCl (pH 7.5 at 37°C), 100 mM NaCl and 1 mM DTT) at 37°C for 30 min.

**Displacement of the Cas9 and Cascade RNP complexes**

100 nM ternary Cas9-crRNA-tracrRNA or 50 nM Cascade-crRNA complex was diluted 1/10 into reaction buffer and pre-incubated at 30°C for 15 min. After pre-incubation heparin was added to a final concentration of 4 mg/ml. Displacement reactions were performed for 20 min at 30°C in reaction buffer: 33 mM Tris-acetate (pH 7.0 at 25°C); 1 nM of $^{33}$P-labelled DNA; 0.1 mg/ml BSA; 1 mM DTT; 2 mM ATP; 150 mM K-acetate; 100 nM MZ-529 (for Cas9) or 50 nM TS-133 (for Cascade); 25 mM phosphocreatine; and 5 U/ml creatine kinase. Reactions were initiated by addition of H.CglI (200 nM) and R.CglI (200 nM) to the reaction mixture and stopped by addition of 1:1 STOP buffer (50 mM EDTA and 10% (v/v) glycerol, pH 8.0 at 25°C). The reaction products were analyzed by electrophoresis using 1.5% (w/v) agarose gels in 40 mM Tris-acetate (pH 8.3 at 25°C), 0.1 mM EDTA at ~8 V/cm for 1h at room temperature (20±5°C). Gels were air-dried and scanned in Fujifilm FLA-5100 fluorescent image analyzer (Fujifilm, Tokyo, Japan) and the image analyzed with OptiQuant software (Packard Instrument) to determine the vomule of each band, taking into account background readings. The fraction of protein-DNA complex in each sample was calculated as $volume_{protein\text{-}DNA}/(volume_{protein\text{-}DNA} + volume_{free\ DNA})$ (1).

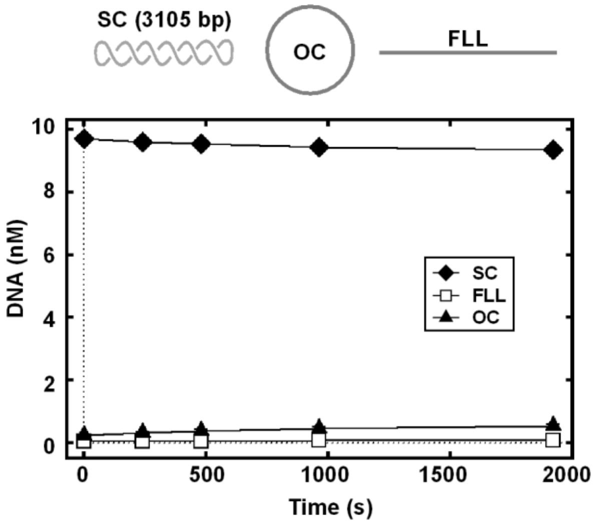


**Supplementary Figure S1. Zero-site plasmid DNA cleavage by CglI.** SC – supercoiled circular DNA, OC – open circle DNA ('nicked'), FLL – full-length linear DNA. Reaction contained 10 nM DNA, 4 mM ATP, 500 nM $R_2H_2$.CglI and was conducted as described in 'Materials and Methods' section. The rate constant value ($1.2 \pm 0.2 \times 10^{-3}$ $s^{-1}$) was obtained by fitting a single exponential to the time-courses of supercoiled form depletion. Points are averages with error bars as standard deviation for at least three repeat reactions.

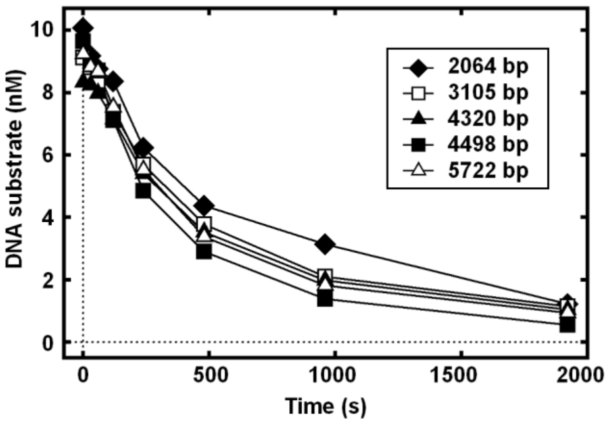


**Supplementary Figure S2. One-site plasmid DNAs with various ring sizes cleavage by CglI.** The ring sizes of one-site circular DNA substrates are indicated in the graph. Reaction contained 10 nM DNA, 4 mM ATP, 500 nM $R_2H_2$.CglI and was conducted as described in 'Materials and Methods' section.

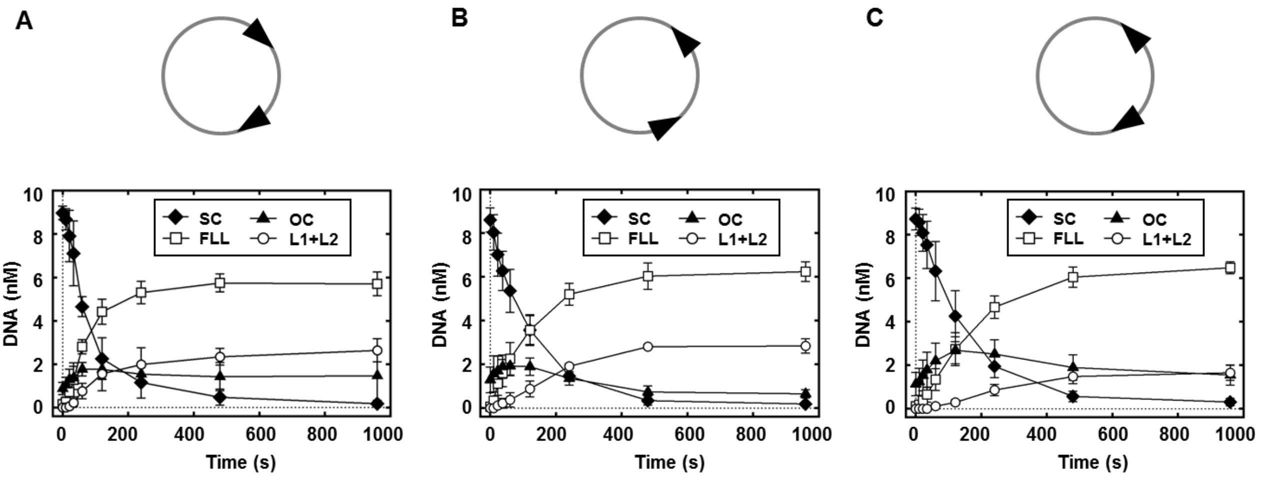


**Supplementary Figure S3. Cleavage of two-site plasmid DNAs by CglI. (A)** Cleavage of two-site HtT1 plasmid DNA. **(B)** Cleavage of two-site HtT2 plasmid DNA. **(C)** Cleavage of two-site TtT plasmid DNA. The CglI recognition sequence (5'-GCCGC-3') is shown as an arrowhead (►). Two-site plasmid DNA substrates are shown above graphs. All reactions contained 10 nM DNA, 4 mM ATP, 500 nM $R_2H_2$.CglI and were conducted as described in 'Materials and Methods' section. The rate constant values ($1.1 \pm 0.1 \times 10^{-2}$ $s^{-1}$ for (A), $7.8 \pm 0.3 \times 10^{-3}$ $s^{-1}$ for (B), $6.4 \pm 0.4 \times 10^{-3}$ $s^{-1}$ for (C)) were obtained by fitting a single exponential to the time-courses of supercoiled form depletion. Points are averages with error bars as standard deviation for at least three repeat reactions.

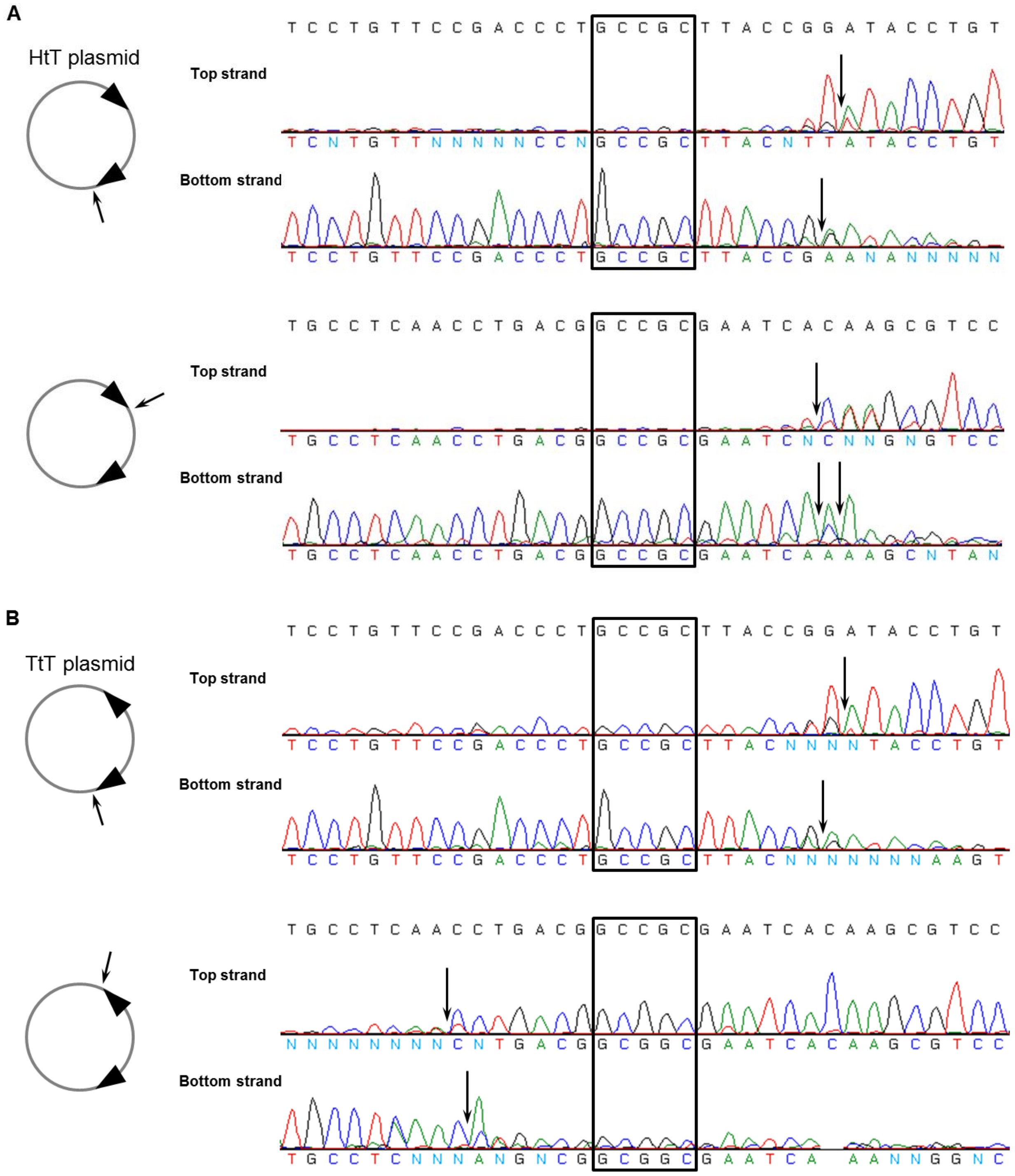


**Supplementary Figure S4. Run-off sequencing to determine the cleavage position of R.CglI.** Determination of cleavage position of HtT **(A)** and TtT **(B)** plasmid DNA at CglI site (black arrowhead ►).The recognition sequence 5'-GCCGC-3' is indicated by the rectangle, the cleavage sites are indicated by arrows. Reactions contained 10 nM DNA, 4 mM ATP, 500 nM $R_2H_2$.CglI and were conducted as described in 'Materials and Methods' section.

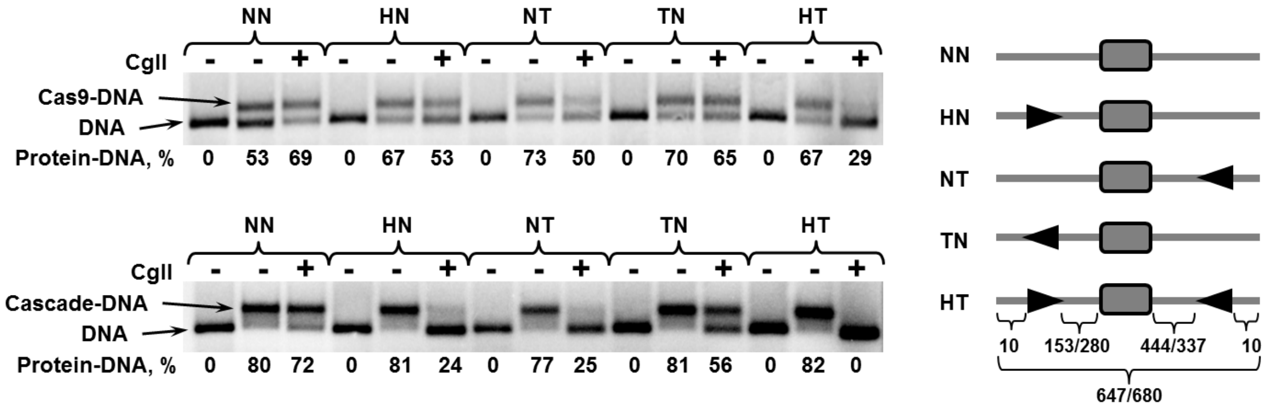


**Supplementary Figure S5. Displacement of Cas9 and Cascade RNP complexes by CglI.** Agarose gels showing Cas9 and Cascade RNP complex displacement from five different DNA substrates (shown in the right). The Cas9 or Cascade RNP complexes with DNA was prepared at 30°C for 15 min and then incubated with CglI at 30°C for 20 min. DNA is shown as a thick line, the CglI recognition sequence (5'-GCCGC-3') is shown as an arrowhead (►), the binding sequences for Cas9 and Cascade RNP complexes are shown as a rectangle, numbers indicate distances in bp for the Cas9 and Cascade DNA fragments, respectively. All reactions contained 1 nM DNA, 2 mM ATP, 200 nM R.CglI, 200 nM H.CglI, 10 nM Cas9 or 5 nM Cascade and were conducted as described in ‘Materials and Methods’ section.

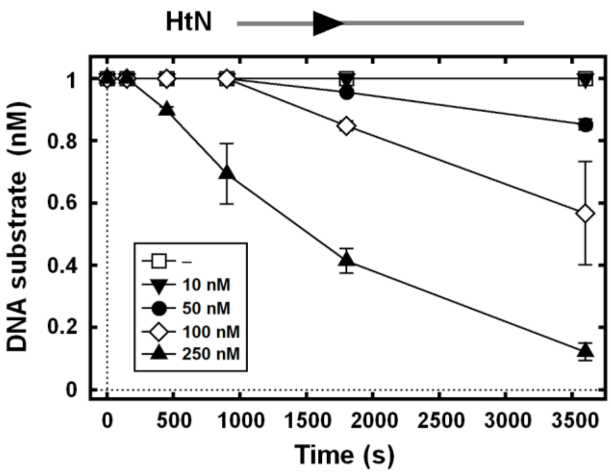


**Supplementary Figure S6. One-site linear DNA cleavage by CglI**. Reactions contained 4320 bp 1 nM HtN linear DNA, the same as used in Figure 3B, 4 mM ATP, 500 nM $R_2H_2$.CglI, 0, 10, 50, 100 or 250 nM S85 DNA *in trans* and were conducted as described in 'Materials and Methods' section. Points are averages with error bars as standard deviation for at least two repeat reactions.

**Supplementary Table S1. Oligonucleotides used in DNA manipulations and experiments.**

| Name | Sequence 5′->3′ | DNA template | Description |
|---|---|---|---|
| Oligonucleotides used for triplex displacement experiments: | | | |
| MZ-503 | CTCCTCGAGCCCGGGTGA | pRMA03_0, pRMA03_1 and pRMA03_1_IN | Generation of 278 bp DNA fragments N, H and T. |
| MZ-504 | CAATTTCACACAGGAAACAGCTATGACC | | |
| MZ-948 | Biotin-CTCCTCGAGCCCGGGTGA | N | Generation of 278 bp 5'-end biotinylated N fragment. |
| MZ-318 | TGGCAGGGCCTGCCGCCC | pECFP-ICAD-S(NLS) | Generation of 85 bp DNA fragment S85. |
| AS-41 | GTTTTGTTGTCTGTCTCACCCCACCCACC GGTTTCGGGTGAAGG | | Generation of 85 bp DNA fragment S85. |
| TFO | TTTCTTTCTTTTCTTCTTTCTT | – | Oligonucleotide recognizing triplex forming sequence in 278 bp DNA fragments N, H and T. |
| Oligonucleotides used for Cas9 and Cascade RNP displacement experiments: | | | |
| MZ-523 | GCTTAACTATGCGGCATCAGAGCAGATTG | pUC-sp3 or pUC19-TS132/133 | Generation of 647 or 680 bp DNA fragment TN. |
| MZ-524 | GCTTAACTATGCCGCATCAGAGCAGATTG | | Generation of 647 or 680 bp DNA fragments HN and HT. |
| MZ-525 | GCTTAACTATGCAGCATCAGAGCAGATTG | | Generation of 647 or 680 bp DNA fragments NN and NT. |
| MZ-526 | GATACCGCTCGCCGCAGCCGAACGACC | | Generation of 647 or 680 bp DNA fragments NT and HT. |
| MZ-528 | GATACCGCTCGCAGCAGCCGAACGACC | | Generation of 647 or 680 bp DNA fragments NN, HN and TN. |
| MZ-529 | GCTGGCGAAAGGGGGATGTG | – | Oligonucleotide complementary to Cas9 crRNA. |
| TS-133 | TTCCCTCAGAACGTATCTGCGCTTATGCG TGGGAGGCCATTGATATAGGTATATTATA TCAAAAAGGGTGGTC | – | Oligonucleotide complementary to Cascade crRNA (5'-GUGAUCCUAUACCUAUA UCAAUGGCCUCCCACGCAU AAGCGUUUUCCCGCACAC GCGGGG-3') (2). |
| 78 nt tracrRNA | GGGCGAAACAACACAGCGAGUUAAAA UAAGGCUUAGUCCGUACUCAACUUGA AAAGGUGGCACCGAUUCGGUGUUUUU | – | RNA for Cas9 RNP complex formation. |
| 42 nt crRNA | CACAUCCCCCUUUCGCCAGCGUUUUA GAGCUGUGUUGUUUCG | – | RNA for Cas9 RNP complex formation. |
| Sequencing oligonucleotides for determination of the CglI cleavage position: | | | |
| MZ-437 | CACTCAAAGGCGGTAATACGG | p2_HtT, p2_TtT | Sequencing oligonucleotide |
| MZ-438 | GAACTCTGTAGCACCGCC | p2_HtT, p2_TtT | Sequencing oligonucleotide |

**Supplementary Table S2. Plasmid DNAs used in DNA manipulations and experiments.**

| Name | Description |
|---|---|
| p0s | 3105 bp zero-site plasmid DNA generated from pMDS36a plasmid (obtained from M. Szczelkun, (3)) by removal of all CglI targets using QuikChange Lightning Multi Site-Directed Mutagenesis Kit (http://www.agilent.com) and recombination using the Tn21 resolvase (4). |
| p1s (p1_3105) | 3105 bp one-site plasmid DNA generated from pMDS36a plasmid (obtained from M. Szczelkun, (3)) by removal of all CglI targets except one (2257) using QuikChange Lightning Multi Site-Directed Mutagenesis Kit (http://www.agilent.com) and recombination using the Tn21 resolvase (4). |
| p1 (p1_4320) | 4320 bp one-site plasmid DNA generated from pMDS36a plasmid (obtained from M. Szczelkun, (3)) by removal of all CglI targets except one (2257) using QuikChange Lightning Multi Site-Directed Mutagenesis Kit (http://www.agilent.com). |
| p1_2064, p1_4498, p1_5722 | 2064, 4498 and 5722 bp one-site plasmid DNAs generated from 4320 bp p1 plasmid by insertion or deletion of DNA fragments with no CglI sites. |
| p2_HtT | 4320 bp two-site HtT (head-to-tail orientation of the asymmetric recognition sequence 5'-GCCGC-3' of CglI) plasmid DNA generated from pMDS36a plasmid (obtained from M. Szczelkun, (3)) by removal of all CglI targets except two (422 and 2257) and invertion of 422 site using QuikChange Lightning Multi Site-Directed Mutagenesis Kit (http://www.agilent.com). |
| p2_HtT2 | 4320 bp two-site HtT2 (head-to-tail orientation of the asymmetric recognition sequence 5'-GCCGC-3' of CglI) plasmid DNA generated from pMDS36a plasmid (obtained from M. Szczelkun, (3)) by removal of all CglI targets except two (422 and 2257) and invertion of 2257 site using QuikChange Lightning Multi Site-Directed Mutagenesis Kit (http://www.agilent.com). |
| p2_TtT | 4320 bp two-site TtT (tail-to-tail orientation of the asymmetric recognition sequence 5'-GCCGC-3' of CglI) plasmid DNA generated from pMDS36a plasmid (obtained from M. Szczelkun, (3)) by removal of all CglI targets except two (422 and 2257) using QuikChange Lightning Multi Site-Directed Mutagenesis Kit (http://www.agilent.com). |
| p2_cat | 3108 & 1212 bp two-site catenane formed from two-site 4320 bp p2_HtT plasmid in the presence of Tn21 resolvase (5). |
| pRMA03_0 | 4865 bp plasmid DNA generated from pRMA03 plasmid (obtained from M. Szczelkun (6)) by removal of the CglI targets at 2220, 2242, 2386, 2389 positions using QuikChange Lightning Multi Site-Directed Mutagenesis Kit (http://www.agilent.com). |
| pRMA03_1 | 4865 bp plasmid DNA generated from pRMA03 plasmid (obtained from M. Szczelkun (6)), by removal of the CglI targets at 2220, 2242, 2386, 2389 positions and introduction of the CglI target (5'-GCCGC-3') at 2620 position using QuikChange Lightning Multi Site-Directed Mutagenesis Kit (http://www.agilent.com). |
| pRMA03_1_IN | 4865 bp plasmid DNA generated from pRMA03 plasmid (obtained from M. Szczelkun (6)), by removal of the CglI targets at 2220, 2242, 2386, 2389 positions and introduction of the CglI target (5'-GCGGC-3') at 2620 position using QuikChange Lightning Multi Site-Directed Mutagenesis Kit (http://www.agilent.com). |
| pECFP-ICAD-S(NLS) | 5556 bp plasmid DNA with triplex forming sequence (obtained from A. Silanskas, (7)). |
| pUC-sp3 | 2726 bp plasmid DNA with Cas9 target sequence (obtained from T. Karvelis, (8)). |
| pUC19-TS132/133 | 2759 bp plasmid DNA with Cascade target sequence (obtained from T. Sinkunas, (2)). |

**Supplementary Table S3. Rate constants of DNA cleavage.**

| Figure | DNA substrate | Rate constants*, ($s^{-1}$) |
|---|---|---|
| 2A | one-site SC | $5.3 \pm 0.3 \times 10^{-3}$ |
| 2B | two-site SC | $1.1 \pm 0.1 \times 10^{-2}$ |
| 2C | two-site SCcat | $1.2 \pm 0.1 \times 10^{-2}$ |
| 3A | one-site SC | $3.6 \pm 0.2 \times 10^{-3}$ |
| | one-site OC | $4.2 \pm 0.7 \times 10^{-3}$ |
| | one-site FLL | $3.0 \pm 0.3 \times 10^{-3}$ |
| 3B | one-site HtN DNA fragment | $2.0 \pm 0.3 \times 10^{-3}$ |
| | two-site HtH DNA fragment | $8.9 \pm 1.9 \times 10^{-4}$ |
| | two-site HtT DNA fragment | $3.3 \pm 0.1 \times 10^{-3}$ |
| 4A | two-site HtT1 DNA fragment | $3.3 \pm 0.1 \times 10^{-3}$ |
| | two-site HtT2 DNA fragment | $4.4 \pm 0.4 \times 10^{-3}$ |
| 4B | two-site HtH DNA fragment | $8.9 \pm 1.9 \times 10^{-4}$ |
| | two-site TtT DNA fragment | $2.0 \pm 0.2 \times 10^{-3}$ |
| S1 | zero-site SC | $1.2 \pm 0.2 \times 10^{-3}$ |
| S3 | two-site HtT plasmid DNA | $1.1 \pm 0.1 \times 10^{-2}$ |
| | two-site HtT2 plasmid DNA | $7.8 \pm 0.3 \times 10^{-3}$ |
| | two-site TtT plasmid DNA | $6.4 \pm 0.4 \times 10^{-3}$ |

*The rate constant values were obtained by fitting a single exponential to the data. The cleavage experiments were repeated three times and the values represent the means and the standard deviations. Rate constants in Figures 2, 4, S1 and S3 refer to the disappearance of substrate; in Figure 3 the rate refers to the appearance of product. SC – supercoiled circular DNA, OC – open circle DNA (‘nicked’), FLL – full-length linear DNA, SCcat – supercoiled circular catenane, HtH – (head-to-head orientation of the asymmetric recognition sequence 5'-GCCGC-3' of CglI), HtT – (head-to-tail orientation of the asymmetric recognition sequence 5'-GCCGC-3' of CglI), TtT – (tail-to-tail orientation of the asymmetric recognition sequence 5'-GCCGC-3' of CglI).

**Supplementary Table S4. Rate constants of TFO displacement.**

| Figure | Reaction | DNA substrate | DNA *in trans* | Rate constants*, ($s^{-1}$) | |
|---|---|---|---|---|---|
| | | | | $k_1$ | $k_2$ |
| 5A | R + ATP | H | – | ≈ 0 | – |
| | H + ATP | H | – | $3.5 \pm 0.7 \times 10^{-5}$ | – |
| | H + R | H | – | $5.0 \pm 3.1 \times 10^{-6}$ | – |
| | H + R + AMP-PNP | H | – | $5.8 \pm 5.9 \times 10^{-6}$ | – |
| | Hmut + R + ATP | H | – | $5.3 \pm 2.4 \times 10^{-6}$ | – |
| | H + Rmut + ATP | H | – | $4.1 \pm 0.1 \times 10^{-2}$ | $4.6 \pm 0.6 \times 10^{-4}$ |
| | H + R + ATP | H | – | $4.5 \pm 0.1 \times 10^{-2}$ | $4.4 \pm 0.5 \times 10^{-4}$ |
| 5B | H + R + ATP | N | – | $2.9 \pm 0.3 \times 10^{-5}$ | – |
| | H + R + ATP | T | – | $9.1 \pm 2.0 \times 10^{-3}$ | $3.3 \pm 0.4 \times 10^{-4}$ |
| | H + R + ATP | H | – | $4.5 \pm 0.1 \times 10^{-2}$ | $4.4 \pm 0.5 \times 10^{-4}$ |
| 6A | H + R + ATP | N | N | $9.6 \pm 1.7 \times 10^{-6}$ | – |
| | H + R + ATP | N | T | $2.6 \pm 0.2 \times 10^{-4}$ | – |
| | H + R + ATP | N | H | $3.0 \pm 0.3 \times 10^{-4}$ | – |
| 6B | H + Rmut + ATP | CN | L0 | $1.7 \pm 0.2 \times 10^{-5}$ | – |
| | H + Rmut + ATP | CN | p0s | $1.3 \pm 0.3 \times 10^{-5}$ | – |
| | H + Rmut + ATP | CN | L1 | $1.3 \pm 0.1 \times 10^{-4}$ | – |
| | H + Rmut + ATP | CN | p1s | $1.6 \pm 0.1 \times 10^{-4}$ | – |
| | H + Rmut + ATP | LN | L0 | $2.4 \pm 0.3 \times 10^{-5}$ | – |
| | H + Rmut + ATP | LN | p0s | $2.9 \pm 0.1 \times 10^{-5}$ | – |
| | H + Rmut + ATP | LN | L1 | $1.8 \pm 0.1 \times 10^{-4}$ | – |
| | H + Rmut + ATP | LN | p1s | $2.1 \pm 0.2 \times 10^{-4}$ | – |
| 7 | H + R + ATP | N (immobilized) | – | $1.3 \pm 0.3 \times 10^{-5}$ | – |
| | H + R + ATP | N (immobilized) | H | $2.0 \pm 0.2 \times 10^{-3}$ | – |
| | H + R + ATP | N (immobilized) | H (immobilized) | $3.2 \pm 0.3 \times 10^{-5}$ | – |

*The rate constants were calculated using a single or double exponential fits to the data. The TFO displacement experiments were repeated three times and the values represent the means and the standard deviations. Rate constants in all figures refer to the disappearance of triplex substrate.

***Supplementary references***


1. Firman, K. and Szczelkun, M.D. (2000) Measuring motion on DNA by the type I restriction endonuclease EcoR124I using triplex displacement. *Embo J*, **19**, 2094-2102.
2. Sinkunas, T., Gasiunas, G., Waghmare, S.P., Dickman, M.J., Barrangou, R., Horvath, P. and Siksnys, V. (2013) In vitro reconstitution of Cascade-mediated CRISPR immunity in Streptococcus thermophilus. *EMBO J*, **32**, 385-394.
3. Peakman, L.J., Antognozzi, M., Bickle, T.A., Janscak, P. and Szczelkun, M.D. (2003) S-adenosyl methionine prevents promiscuous DNA cleavage by the EcoP1I type III restriction enzyme. *J Mol Biol*, **333**, 321-335.
4. Szczelkun, M.D. and Halford, S.E. (1996) Recombination by resolvase to analyse DNA communications by the SfiI restriction endonuclease. *Embo J*, **15**, 1460-1469.
5. Peakman, L.J. and Szczelkun, M.D. (2004) DNA communications by Type III restriction endonucleases--confirmation of 1D translocation over 3D looping. *Nucleic Acids Res*, **32**, 4166-4174.

6. Smith, R.M., Josephsen, J. and Szczelkun, M.D. (2009) The single polypeptide restriction-modification enzyme LlaGI is a self-contained molecular motor that translocates DNA loops. *Nucleic Acids Res*, **37**, 7219-7230.
7. Silanskas, A., Foss, M., Wende, W., Urbanke, C., Lagunavicius, A., Pingoud, A. and Siksnys, V. (2011) Photocaged variants of the MunI and PvuII restriction enzymes. *Biochemistry*, **50**, 2800-2807.
8. Sapranauskas, R., Gasiunas, G., Fremaux, C., Barrangou, R., Horvath, P. and Siksnys, V. (2011) The Streptococcus thermophilus CRISPR/Cas system provides immunity in Escherichia coli. *Nucleic Acids Res*, **39**, 9275-9282.
